# Arabidopsis CaM Binding Protein CBP60g Contributes to MAMP-Induced SA Accumulation and Is Involved in Disease Resistance against *Pseudomonas syringae*

**DOI:** 10.1371/journal.ppat.1000301

**Published:** 2009-02-13

**Authors:** Lin Wang, Kenichi Tsuda, Masanao Sato, Jerry D. Cohen, Fumiaki Katagiri, Jane Glazebrook

**Affiliations:** 1 Department of Plant Biology, Microbial and Plant Genomics Institute, University of Minnesota, St. Paul, Minnesota, United States of America; 2 Department of Life Sciences, Graduate School of Arts and Sciences, The University of Tokyo, Meguro-ku, Tokyo, Japan; 3 Department of Horticultural Science, Microbial and Plant Genomics Institute, University of Minnesota, St. Paul, Minnesota, United States of America; Massachusetts General Hospital, United States of America

## Abstract

Salicylic acid (SA)-induced defense responses are important factors during effector triggered immunity and microbe-associated molecular pattern (MAMP)-induced immunity in plants. This article presents evidence that a member of the Arabidopsis CBP60 gene family, *CBP60g*, contributes to MAMP-triggered SA accumulation. *CBP60g* is inducible by both pathogen and MAMP treatments. *Pseudomonas syringae* growth is enhanced in *cbp60g* mutants. Expression profiles of a *cbp60g* mutant after MAMP treatment are similar to those of *sid2* and *pad4*, suggesting a defect in SA signaling. Accordingly, *cbp60g* mutants accumulate less SA when treated with the MAMP flg22 or a *P. syringae hrcC* strain that activates MAMP signaling. MAMP-induced production of reactive oxygen species and callose deposition are unaffected in *cbp60g* mutants. CBP60g is a calmodulin-binding protein with a calmodulin-binding domain located near the N-terminus. Calmodulin binding is dependent on Ca^2+^. Mutations in CBP60g that abolish calmodulin binding prevent complementation of the SA production and bacterial growth defects of *cbp60g* mutants, indicating that calmodulin binding is essential for the function of CBP60g in defense signaling. These studies show that CBP60g constitutes a Ca^2+^ link between MAMP recognition and SA accumulation that is important for resistance to *P. syringae*.

## Author Summary

Plants respond to attack by microbial pathogens through activation of a battery of defense responses. This activation is controlled by a complex signaling network. Disease resistance depends on rapid activation of plant defense responses. Improved understanding of the signaling network may lead to development of crops with improved disease resistance. Here, we used the model plant Arabidopsis thaliana to study activation of defense responses after infection by a bacterial pathogen, *Pseudomonas syringae*. We found that a gene not previously known to function in defense signaling, *CBP60g*, is needed for resistance. By studying plants with mutations in this gene, we found that *CBP60g* contributes to the increases in levels of the important signaling molecule, salicylic acid, that occur after pathogen recognition. We also found that the CBP60g protein binds calmodulin, a protein that mediates calcium regulation of protein function. Calmodulin binding was necessary for the function of CBP60g in disease resistance. We conclude that CBP60g is a protein that mediates calmodulin-dependent activation of salicylic acid signaling in response to pathogen recognition.

## Introduction

Plant innate immunity is multi-layered and tightly regulated by a complex signaling network [1]. Defense against biotrophic or hemibiotrophic bacterial pathogens can be thought of as consisting of two branches: the broad and nonspecific defenses triggered by the perception of microbe- or pathogen-associated molecular patterns (MAMPs or PAMPs), and the robust and relatively more specific resistance mediated by resistance (R) genes [2],[3]. MAMPs are proteins and other molecules characteristic of microbes. MAMP-triggered defense is initiated by perception of MAMPs by pattern-recognition receptors (PRRs). Well-characterized examples in Arabidopsis include recognition of flagellin by the receptor kinase FLS2 [4], of Ef-Tu by the receptor kinase EFR [5], and of chitin by the LysM receptor kinase CERK1. Direct binding has been demonstrated for FLS2 and EFR, but not for CERK1 [6],[7]. FLS2 and EFR require a second kinase, BAK1, to initiate defense signaling [8]–[10]. Signaling activation results in an oxidative burst produced by the NADPH oxidase encoded by AtrbohD, which is in turn required for deposition of callose at the cell wall [11]. Other responses include closure of stomata, activation of a MAP kinase cascade, and a suite of gene expression changes [12]–[14]. MAMP responses are effective in limiting pathogen growth, as pre-treatment with flg22, a peptide derived from flagellin, dramatically reduces growth of *Pseudomonas syringae* pv. *tomato* DC3000 (*Pst* DC3000) in an FLS2-dependent manner [15], *efr* plants are more susceptible to *Agrobacterium tumefaciens* [5], and *cerk1* mutants are more susceptible to *Alternaria brassicicola* [6],[7].

Bacterial pathogens produce numerous virulence effector proteins that are secreted into the host cytoplasm, where many of them disrupt plant defense responses [2],[3],[16]. Plants can counter this if they have one or more appropriate Resistance (R) genes. R proteins detect effectors by directly binding effector proteins or by sensing the cellular disturbance caused by effector activity [17]. R protein activation results in induction of additional layers of defenses, including production of reactive oxygen species (ROS) and activation of the hypersensitive response (HR), a programmed cell death response thought to limit pathogen access to water and nutrients [18]. R gene recognition of an effector also results in activation of the salicylic acid (SA)-dependent defense signaling pathway, which plays an important role in resistance [19].

Several components of the SA signaling circuitry have been identified through genetic analysis in Arabidopsis. ENHANCED DISEASE SUSCEPTIBILITY 1 (EDS1) and PHYTOALEXIN DEFICIENT 4 (PAD4) are physically-interacting proteins that are required for SA synthesis in response to some, but not all, pathogens [20]–[23]. PAD4 and EDS1 are also required for pathogen-induced expression of many SA-independent genes [24]. SALICYLIC ACID INDUCTION DEFICIENT 2 (SID2), which encodes isochorismate synthase, and ENHANCED DISEASE SUSCEPTIBLITY 5 (EDS5) are required for SA synthesis [25],[26]. In response to elevated SA, NONEXPRESSOR OF PR GENES 1 (NPR1) undergoes a transition from oligomer to monomer and translocates to the nucleus [27],[28]. Once there, it interacts with transcription factors to modulate expression of defense genes such as PATHOGENESIS RELATED 1 (PR1) [29],[30].

Recent studies have shown that SA signaling is an integral part of the MAMP response, as well as of R-gene mediated resistance. Treatment with the MAMPs flg22 or bacterial lipopolysaccharide (LPS) caused SA accumulation and systemic acquired resistance, a systemic response associated with SA [31]. Flg22 treatment also induced many canonical SA-related genes, including *SID2*, *EDS5*, *NPR1*, and *PR1* [32]. SA was produced in response to flg22 or challenge with *Pst* DC3000 *hrcC*, a strain that is unable to transport effectors and thus serves as an elicitor of the MAMP response [33]. Many gene expression changes caused by challenge with *Pst* DC3000 *hrcC* were reduced in *pad4* or *sid2* mutants, demonstrating that MAMP-induced SA plays a role in the MAMP response [33]. Importantly, resistance to *Pst* DC3000 induced by pre-treatment with flg22 was compromised in *pad4* and *sid2* mutants, demonstrating that MAMP-induced SA is important for MAMP-triggered resistance [33]. The nature of the link between MAMP recognition and activation of SA signaling remains to be determined.

Calcium signaling is another aspect of plant defense responses that has been implicated in both MAMP-triggered and R-gene mediated resistance responses. Rapid influxes of cytosolic Ca^2+^ have been observed after treatment of *Nicotiana plumbaginifolia* cells with MAMPs such as LPS, oligogalacturonides (OGs), and cryptogein, a small protein from *Phytophthora cryptogea* that elicits defense responses and cell death in tobacco [34]. In Arabidopsis, peptidoglycan from gram-positive bacteria acted as a MAMP and induced cytosolic Ca^2+^ influx, as did flg22 [35]. In the case of treatment of *Nicotiana plumbaginifolia* cells with cryptogein, blocking calcium influx with La^3+^ blocked downstream responses including MAP kinase activation, gene expression changes, and the HR, indicating that Ca^2+^ influx is required for these responses [34]. In Arabidopsis, production of NO in response to LPS required a Ca^2+^ influx that depended on the CYCLIC NUCLEOTIDE GATED ION CHANNEL 2 (CNGC2) [36], and flg22 treatment resulted in calcium-dependent phosphorylation of a syntaxin [37]. Calcium signaling also plays a role in R-gene mediated responses. Cytosolic calcium increased in response to treatment of Arabidopsis carrying the R gene *RPM1* with *P. syringae* expressing the cognate effector protein *avrRpm1*. Blocking calcium influx with La^3+^ blocked the hypersensitive response characteristic of this resistant interaction [38]. A mutation in CNGC2 called *dnd1* blocks the HR in several cases of Arabidopsis R-gene mediated resistance [39], suggesting that CNGC2 may be generally important for calcium influx during defense responses.

Calcium signals can be transduced by binding of calcium to calmodulin (CaM), a ubiquitous small calcium-binding protein. Binding of CaM to other proteins modulates their activities [40]. The barley MLO protein is a CaM-binding protein that acts as a repressor of defense responses. Mutations that prevent CaM binding reduce the repressing activity of the protein [41]. A number of CaM-binding proteins that are pathogen-inducible have been identified, suggesting that they may participate in the defense response [40],[42],[43]. These include members of the CBP60 family in Arabidopsis [44]. The AtCBP60 family consists of seven members (from AtCBP60a to AtCBP60g: At5g62570; At5g57580; At2g18750; At4g25800; At2g24300; At4g31000; At5g26920; Figure S1) that were identified based on their protein sequence similarities to tobacco and maize homologues [45]–[47]. Domains that bind CaM in a Ca^2+^ dependent manner have been mapped to the C-terminal ends of five family members [44]. AtCBP60 genes were shown to be differentially expressed in response to bacterial pathogens and inducers of defense responses but their biological functions remain unknown [43].

We have studied a member of the Arabidopsis CBP60 CaM-binding protein family, CBP60g (At5g26920), which lacks the C-terminal CaM-binding domain of other family members. We found that it is inducible by infection with *Pseudomonas syringae* pv. *maculicola* strain ES4326 (*Psm* ES4326) and by MAMPs. Loss-of-function mutants allowed enhanced growth of *Psm* ES4326, demonstrating a role of this protein in disease resistance. Characterization of mutant lines revealed a defect in SA signaling following MAMPs treatment, indicating a role for CBP60g in activation of SA signaling by MAMPs. We demonstrated that CBP60g binds CaM, and determined that the CaM-binding domain lies in the N-terminal part of the protein. Mutant proteins that lacked CaM-binding activity failed to complement the defense defects of a *cbp60g* loss-of-function mutant, indicating that CaM binding is important for the function of CPB60g in defense signaling.

## Results

### AtCBP60g Expression is Induced in Response to Pathogen Attack and MAMPs

**Figure 1:**
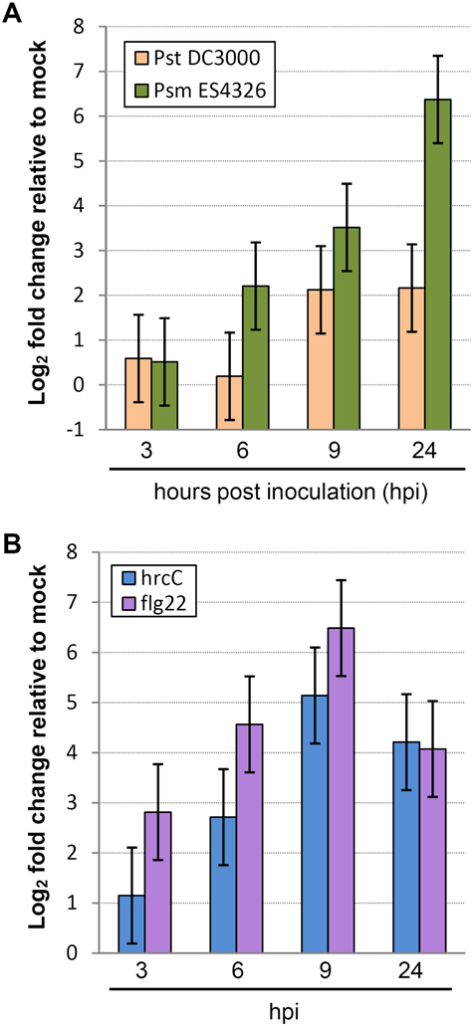
Changes in *CBP60g* expression levels after pathogen or MAMP inoculation. Each bar represents the log_2_ ratio of the mean expression value in treated plants relative to mock-treated plants. Data were normalized using the control gene *ACTIN2*. Data were obtained in three independent experiments, each with two technical replicates, and analyzed by ANOVA. Error bars represent standard error. (A) CBP60g expression in response to inoculation with *Psm* ES4326 or *Pst* DC3000 inoculation. (B) CBP60g expression in response to *Pst* DC3000 *hrcC* or flg22 inoculation.

We noticed that, according to microarray data, Arabidopsis *CBP60g* (CaM-binding protein 60-like.g; At5g26920) was strongly up-regulated in response to infection by the virulent strain *Psm* ES4326 [48]. We used the real-time quantitative polymerase chain reaction (qRT-PCR) to monitor expression of this gene. Figure 1A shows that expression of *CBP60g* was induced between three and six hours after *Psm* ES4326 infection, and expression remained high for at least 24 hours. *CBP60g* expression was also induced between six and nine hours after infection by *P. syringae* pv. *tomato* strain DC3000 (*Pst* DC3000), but to a lesser extent. We further investigated *CBP60g* expression after MAMP treatments. We inoculated wild-type plants with *Pst* DC3000 *hrcC*, a strain defective in delivery of type-III effectors [49]. By three hours after inoculation, and continuing for at least 24 hours, *CBP60g* transcript levels were higher than in mock-treated controls. Infiltration with the purified MAMP, flg22, had an even stronger effect (Figure 1B). These results indicate that expression of *CBP60g* is induced in response to bacterial pathogens and MAMPs.

### Mutations in AtCBP60g Result in Enhanced Susceptibility to *P. syringae*

**Figure 2:**
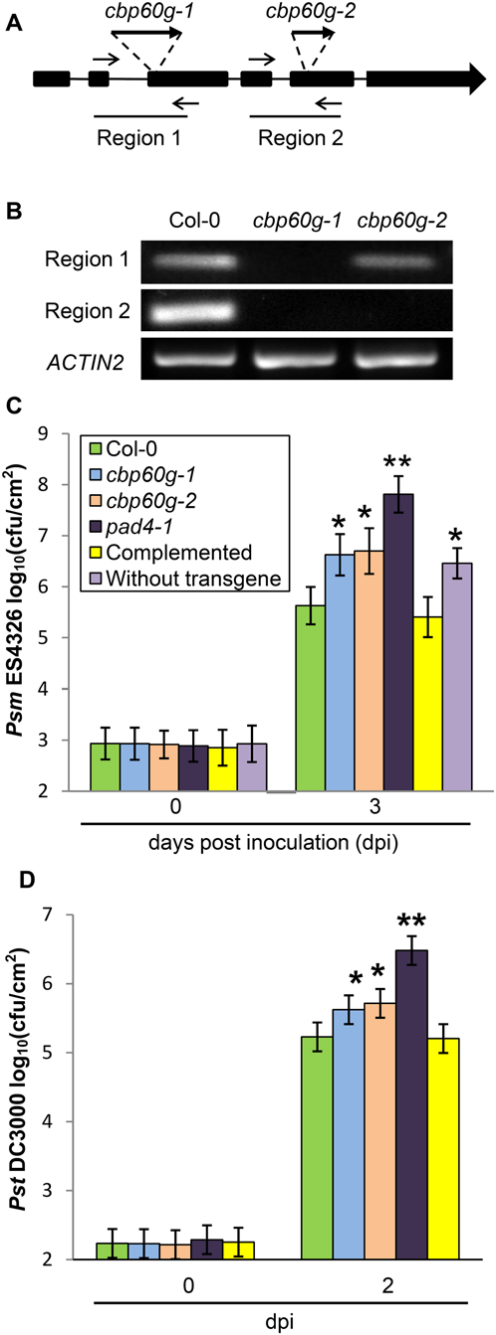
Mutants of *CBP60g* support more bacterial growth than wild-type plants. (A) Illustration of *CBP60g* mutants *cbp60g-1* and *cpb60g-2*. Bold lines, exons; thin lines, introns; bold arrows: T-DNA insertions; thin arrows, primers used for RT-PCR. (B) RT-PCR results showing two regions of the *CBP60g* transcript. (C) Bacterial growth assays using *Psm* ES4326. Each bar at 0 hours or 72 hours represents data from 4 or at least 16 replicates, respectively. Error bars represent standard deviation from 16 samples. Asterisks, p<0.05. P values were calculated using the two-tailed Mann-Whitney U-test. Similar results for the *cbp60g* mutants were obtained in two other independent experiments. Complemented, *cbp60g-1* plants carrying wild-type CBP60g as a transgene; Without transgene; siblings of the complemented plants lacking the transgene.

We studied the function of *CBP60g* using loss-of-function mutants. We acquired two T-DNA insertion mutants of *CBP60g*, *SALK_023199* and *GABI_075G12*, and named them *cbp60g-1* and *cbp60g-2*, respectively. According to the SIGnAL database (http://signal.salk.edu/), the T-DNA insertion of *cbp60g-1* is located in the third exon of At5g26920, while in *cbp60g-2* it is in the fifth exon, as shown in Figure 2A. Reverse transcription PCR (RT-PCR) showed that the *CBP60g* transcript was absent in *cbp60g-1* homozygotes and only partial in *cbp60g-2* homozygotes (Figure 2B), suggesting that neither mutant allele produces functional CBP60g protein.

To test *cbp60g* mutants for enhanced susceptibility to *P. syringae*, wild type (Col-0), *cbp60g-1*, and *cbp60g-2* plants were inoculated with *Psm* ES4326, and bacterial titer was determined three days later. Figure 2C shows that both mutant lines supported significantly more bacterial growth than wild-type plants, but less than the extremely susceptible *pad4* plants [22]. The fact that two independent mutations in *CBP60g* result in similar enhanced susceptibility phenotypes strongly suggests that these phenotypes result from mutations in *CBP60g*. This idea was further verified by introducing a genomic clone containing *CBP60g* and its promoter (1093 base pairs upstream of its start codon) into homozygous *cbp60g-1* plants. The progeny of a transformant that was hemizygous for the transgene were infected with *Psm* ES4326 and bacterial titers in individual plants were determined three days later. The average titer in plants carrying the wild-type transgene was similar to wild-type plants, while the average titer in sibling plants lacking the transgene was significantly higher and similar to untransformed *cbp60g-1* homozygotes. *Pst* DC3000 also grew to higher titers in *cbp60g* mutants than in wild-type plants, and this phenotype was also complemented by a wild-type *CBP60g* transgene as shown in Figure 2D. Based on these experiments, we conclude that *CBP60g* is required for wild-type levels of resistance to both *Psm* ES4326 and *Pst* DC3000.

### Expression Profiling of *cbp60g-1* Suggests a Defect in MAMP-Triggered SA Signaling

In an effort to understand how *cbp60g* mutations affect defense responses against bacterial pathogens, we conducted microarray profiling experiments using a customized long-oligonucleotide microarray with probes for 464 pathogen-responsive genes, representing diverse expression patterns [50]. Expression profiling and data analysis using the custom microarray were carried out as described in Methods. First, we compared wild-type and homozygous *cbp60g-1* plants 24 hours after inoculation with *Psm* ES4326. Other than *CBP60g* itself, there was only one gene (*COR47*, At1g20440) that was significantly different from wild-type by more than two-fold (Table S1). These results indicated that *CBP60g* did not have a major effect on gene expression 24 hours after *Psm* ES4326 infection.

Since *CBP60g* is also inducible by MAMP treatments, we tested the *cbp60g-1* mutant for alterations in gene expression following inoculation with *Pst* DC3000 *hrcC*. Wild-type and *cbp60g-1* plants were mock-inoculated or inoculated with *Pst* DC3000 *hrcC*, and samples were collected after three and nine hours, when MAMP-triggered responses generally occur [51],[52]. At three hours after inoculation with *Pst* DC3000 *hrcC*, 31 genes showed differential expression between wild-type and *cbp60g-1* plants (q<0.05) as shown in Table S2. At nine hours, 43 genes were differentially expressed (q<0.05). Clearly, the effect of CBP60g on gene expression changes during a MAMP response is larger than it is 24 hours after *Psm* ES4326 inoculation.

**Figure 3:**
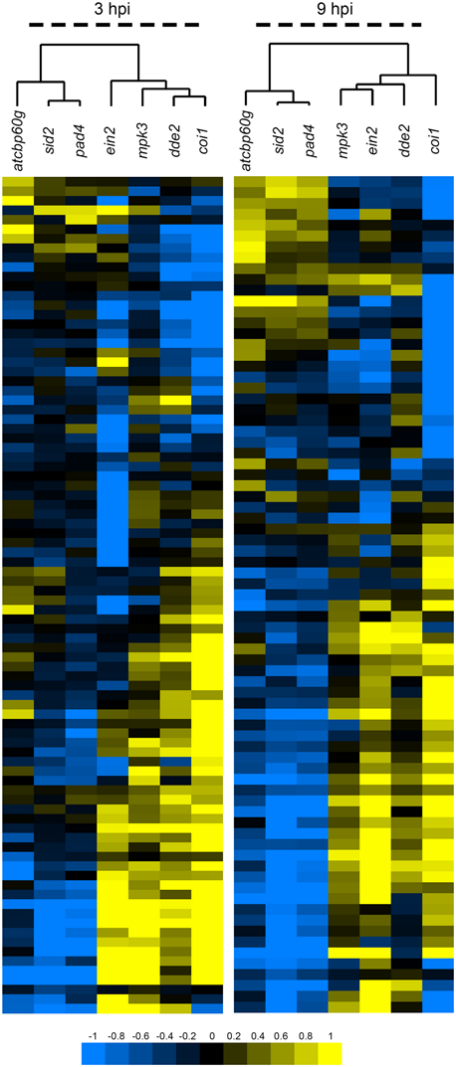
Expression patterns identified by agglomerative hierarchical clustering. The log_2_ ratios of each indicated sample comparison were used for the analysis. Clustering was separately performed at each time point with Cluster [58] using the uncentered Pearson correlation and complete linkage clustering. Results were visualized with Treeview [58]. Blue indicates negative values, yellow positive values and black zero, as shown on the color scale at the bottom of the figure.

To determine in which sector of the defense signaling network CBP60g acts, we compared the effects of *cbp60g-1* on the response to DC3000 *hrcC* to the effects of other mutations that perturb the defense signaling network. We chose *pad4* and *sid2*, which reduce SA signaling [26],[53]; *coi1* and *dde2*, which reduce JA signaling [54],[55]; *ein2*, which reduces ethylene signaling [56], and *mpk3*, which may affect MAMP signaling [57]. Wild-type and mutant plants were inoculated with *Pst* DC3000 *hrcC* and wild-type plants were also mock-inoculated. Samples were again collected after three and nine hours. We selected genes with significantly different expression levels in at least one of the seven mutants compared to wild-type, after *Pst* DC3000 *hrcC* inoculation (q<0.05; Table S2). Among these, we further selected genes that were induced or repressed by at least two-fold in wild-type plants inoculated with *Pst* DC3000 *hrcC* compared to mock-inoculated wild-type plants. For the 88 genes that met these conditions at the three hour time point, the log_2_ ratios of *cbp60g* to Col-0, *coi1-1* to Col-0, *dde2-2* to Col-0, *ein2-1* to Col-0, *mpk3* to Col-0, *pad4-1* to Col-0, and *sid2-2* to Col-0 were subjected to complete-linkage agglomerative hierarchical clustering [58]. The same procedure was carried out on the 77 genes that met these conditions at the nine hour time point. Figure 3 shows that the effects of *cbp60g* most closely resembled those of *sid2* and *pad4*, which disrupt SA signaling during the MAMP response. At nine hours, the correlations between the *cbp60g* to Col log_2_ ratios and the *pad4* to Col and *sid2* to Col log_2_ ratios were 0.75 and 0.68, respectively as shown in Table 1. As PAD4 and SID2 function in SA signaling, these strong correlations between the effects of *cbp60g* and those of mutations known to disrupt SA signaling suggested that CBP60g functions in activation of SA signaling during the MAMP response.

**Table 1:** Correlation coefficients between expression profiles at 9 hpi after *Pst* DC3000 *hrcC* treatment.

	*cbp60g-1*	*coi1*	*dde2*	*ein2*	*mpk3*	*pad4*	*sid2*
***cbp60g-1***	1	−0.52	−0.17	−0.48	−0.45	0.75	0.68
***coi1***	−0.52	1	0.23	0.58	0.44	−0.53	−0.42
***dde2***	−0.17	0.23	1	0.49	0.49	−0.19	−0.11
***ein2***	−0.48	0.58	0.49	1	0.61	−0.56	−0.54
***mpk3***	−0.45	0.44	0.49	0.61	1	−0.52	−0.50
***pad4***	0.75	−0.53	−0.19	−0.56	−0.52	1	0.91
***sid2***	0.68	−0.42	−0.11	−0.54	−0.50	0.91	1

### *SID2* Expression and Free SA Levels Are Reduced in *cbp60g* Mutants

**Figure 4:**
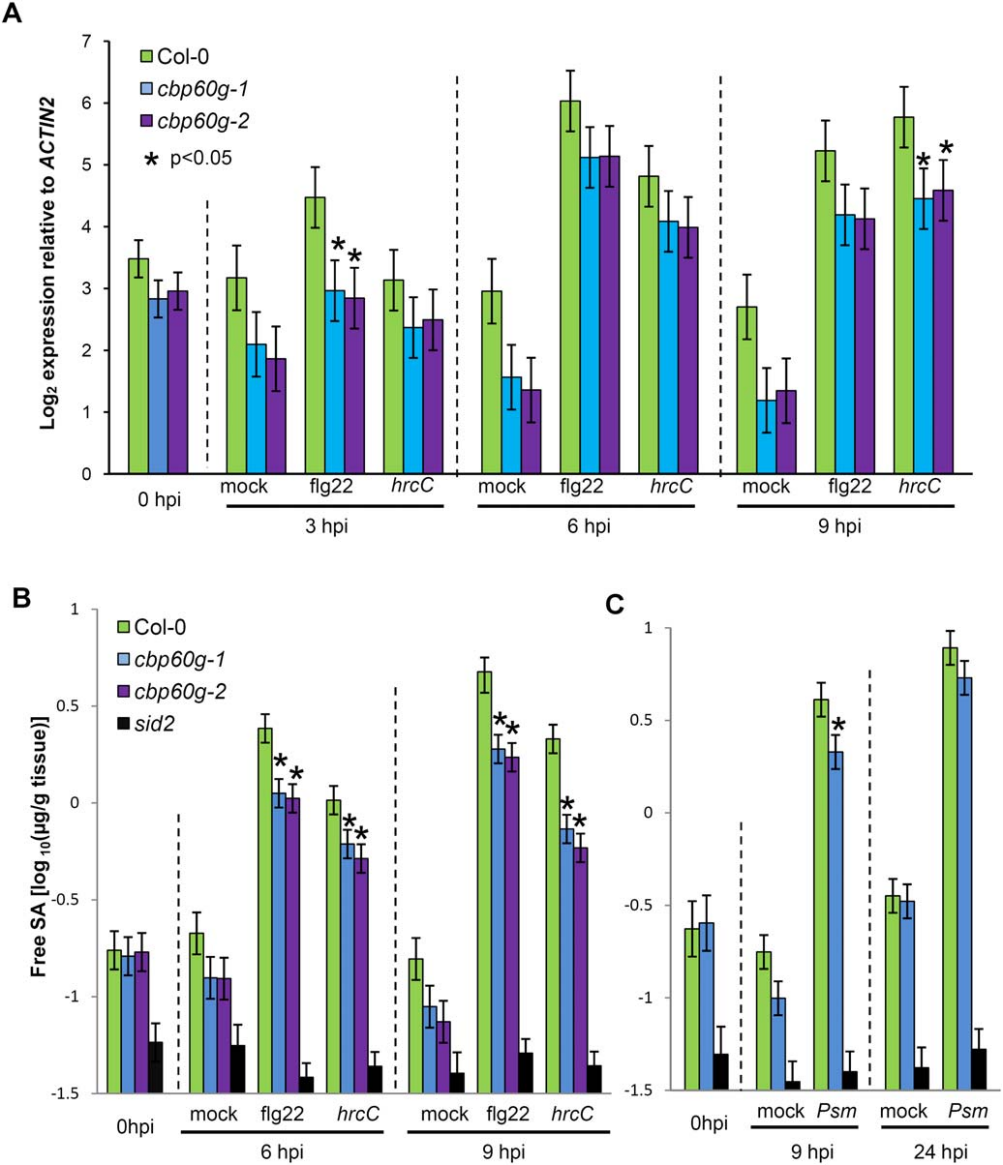
*SID2* expression and SA accumulation in *cbp60g* mutants. (A) *SID2* expression in Col-0 and *cbp60g* mutants after flg22 or *Pst* DC3000 *hrcC* (*hrcC*) treatment. Each bar represents the log_2_ expression value relative to *ACTIN2*. Data was obtained in three independent experiments, each with two technical replicates, analyzed by ANOVA. Error bars represent standard error. (B) Measurement of free SA after flg22 or *Pst* DC3000 *hrcC* treatments. (C) Measurement of free SA after *Psm* ES4326 treatment. For B and C, data from two independent experiments, each consisting of one sample of each type, was analyzed by ANOVA. Error bars represent standard error.

The microarray data also revealed that *SID2* was induced by *Pst* DC3000 *hrcC* inoculation in wild-type plants (1.74-fold at three hours and 3.02-fold at nine hours), and that this induction was attenuated in *cbp60g* mutant plants (the ratio of *SID2* expression in *cbp60g-1* to wild-type is 0.34 at three hours and 0.38 at nine hours). The qRT-PCR results shown in Figure 4A confirmed that *SID2* expression was induced by DC3000 *hrcC* inoculation and flg22 treatment, as we have reported previously [33]. *SID2* expression was reduced in both *cbp60g* mutants, with statistically significant differences observed three hours after flg22 treatment and nine hours after DC3000 *hrcC* inoculation. Since SID2 is required for SA synthesis during the defense response, we suspected that SA accumulation was also compromised in *cbp60g* mutants.

To determine whether SA levels were lower in *cbp60g* mutants, we measured free (non-conjugated) SA levels in wild-type, *cbp60g*, and *sid2* plants following mock treatment, flg22 treatment, and DC3000 *hrcC* inoculation. Figure 4B shows that SA levels in both *cbp60g* mutants were significantly lower than in wild-type plants at six and nine hours following flg22 treatment and at nine hours following DC3000 *hrcC* inoculation (note the log_10_ scale). SA levels in *sid2* plants were very low and did not respond to treatments. We also measured free SA levels in *cbp60g-1* following inoculation with *Psm* ES4326 or the avirulent strain *Psm* ES4326 *avrRpt2*. After *Psm* ES4326 inoculation, the SA level in *cbp60g-1* was only lower than in wild-type plants at nine hours after inoculation (q = 0.002) but not 24, and the extent of the reduction at 9 hours was less than in the case of flg22 or *Pst* DC3000 *hrcC* treatments (Figure 4C). To verify that the SA difference we observed in *Psm* ES4326-inoculated plants was not due to enhanced bacterial growth in the *cbp60g-1* mutant, we monitored bacterial titers in the plants used for SA extraction. As shown in Figure S2, no significant differences in titer were observed among wild type and *cbp60g-1* mutants at 9 or 24 hours after inoculation. After *Psm* ES4326 *avrRpt2* inoculation, there were no significant differences (q<0.05) in SA accumulation between wild-type and *cbp60g* mutants (Figure S3). Taken together, these results show that CBP60g contributes to SA accumulation during the MAMP response and at early times during attack by *Psm* ES4326.

### CBP60g Mutants Do Not Affect the flg22-Triggered ROS Burst, Callose Deposition, or flg22 Inhibition of Seedling Growth

Having observed that *cbp60g* mutants were deficient in MAMP-induced SA accumulation, we tested *cbp60g* mutants for defects in other MAMP-triggered responses. Three characteristic MAMP signaling responses are transient production of reactive oxygen species (ROS), deposition of callose, and inhibition of seedling growth [51]. We monitored flg22-induced ROS production in wild-type, *cbp60g-1*, *cbp60g-2*, and *fls2* plants. *FLS2* encodes the flagellin receptor, thus *fls2* mutants do not respond to flg22. There was no difference in production of ROS between wild-type plants and *cbp60g* mutants, while ROS production was abolished in *fls2* plants (Figure S4). Callose deposition at twelve hours after flg22 treatment was assayed by aniline blue staining and image analysis. No significant differences were observed among wild type and *cbp60g* mutants (Figure S5). No callose deposition was observed in *pmr4* mutants, which lack a callose synthase [59]. Clearly, *cbp60g* mutants are not defective in flg22-induced ROS production or callose deposition. Seedling growth is inhibited by flg22. We tested wild-type, *cbp60g*, *pad4*, *sid2*, and *fls2* seedlings for inhibition by flg22. We found that *cbp60g*, *pad4*, and *sid2* plants all showed growth inhibition similar to wild-type plants, while *fls2* mutants showed very little growth inhibition (Figure S6). Thus, mutations that reduce MAMP-induced SA production do not have a major effect on inhibition of seedling growth by flg22.

### CBP60g Is a CaM Binding Protein with the Calmodulin Binding Domain Located at the N Terminus

**Figure 5:**
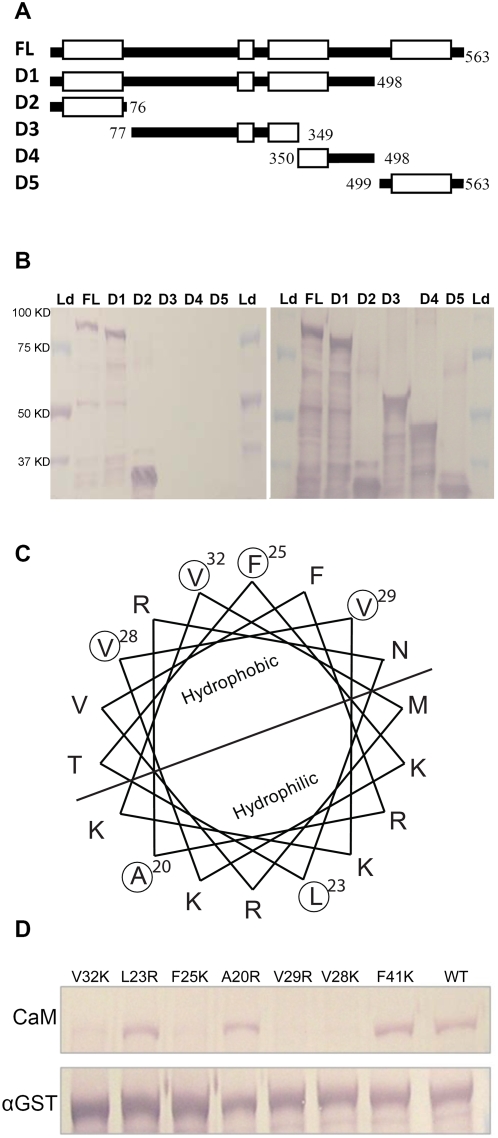
Mapping and site-directed mutagenesis of the CBP60g CaM-binding domain. (A) Illustration of wild-type and deletion constructs of CBP60g protein sequences. FL, full-length wild-type protein; D1 to D5, five deletion constructs. Empty white bars, predicted coiled-coil domains; dark solid bars, non-coiled coil domains. Numbers indicate amino acid positions in the full-length protein. (B) CaM binding by GST-tagged CBP60 deletion constructs. Left, detection of CaM biding; right, detection of GST; Ld, protein size marker ladder. The predicted protein sizes of GST-fusion deletion constructs for FL, D1, D2, D3, D4, and D5 are 92.3 KD, 89.1 KD, 37.6 KD, 63.1 KD, 48.1KD, and 35.8 KD respectively. (C) Helical wheel projection of the CBP60g CaM-binding domain. Amino acids selected for mutagenesis are circled. (D) CaM binding assay of mutated GST-tagged CBP60g proteins. Upper picture, CaM binding; lower picture, detection of GST.

Five of the eight CBP60 proteins have a CaM binding domain (CBD) at the C terminus [44]. However, the corresponding sequence of CBP60g is poorly conserved (Figure S1). In order to test whether CBP60g binds to CaM and identify possible CBD domain(s) of CBP60g, we predicted its coiled coil domains using the PredictProtein algorithm [60], as this protein secondary structure is shared by nearly all known CBDs [61]. Figure 5A shows the positions of the predicted coiled coil domains.

We tested the ability of CBP60g to bind CaM by constructing a GST-CBP60g protein fusion and expressing it in *Escherichia coli*. Western blotting with anti-GST antibody showed that a protein of the expected molecular weight (approximately 89 kilodaltons) was produced. A replicate blot was incubated with biotinylated CaM. Bound CaM was then detected with streptavidin-conjugated alkaline phosphatase. Figure 5B shows that full-length CBP60g protein bound to CaM. No binding was observed in the absence of Ca^2+^ (Figure S7).

**Figure 6:**
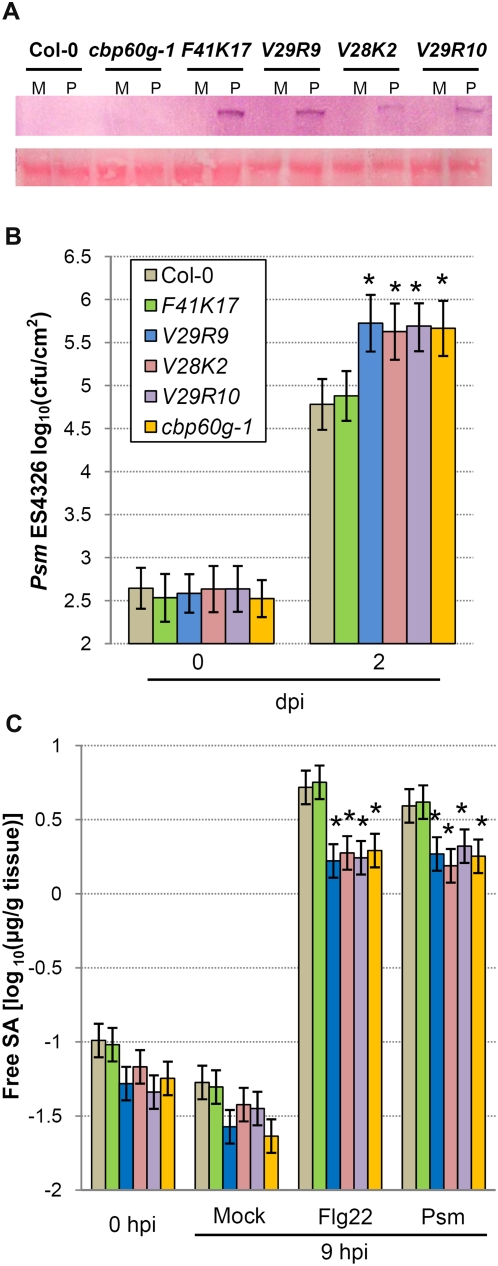
Measurement of bacterial growth and free SA in *cbp60g* transgenic lines. (A) Presence of modified CBP60g proteins in the *cbp60g-1* background. Upper panel shows the immunoblot results using anti-c-Myc antibody; lower panel shows the large subunit of the Ribulose-1,5-bisphosphate carboxylase/oxygenase (Rubisco) stained with Ponceau S as a measurement of the total protein loaded onto each lane. M indicates mock treated, P indicates *Psm* ES4326 treated. (B) Bacterial growth assays using *Psm* ES4326. Each bar at 0 and 48 hours represents 4 or 16 replicates, respectively. Error bars represent standard deviation. P values were calculated using two-tailed Mann-Whitney U-test. Asterisks indicate p<0.05. (C) Measurement of free-SA after flg22 and *Psm* ES4326 treatment. Data were pooled from two independent experiments. Samples were extracted from six leaves for each genotype in each replicate. Error bars represent standard error calculated by ANOVA. Asterisks indicate p<0.01.

We then tested various CBP60g deletion mutants (Figure 6A) in an effort to locate the CaM binding domain (CBD). Figure 6B shows that a 76 amino acid fragment from the N-terminus of the protein was sufficient for CaM binding. Further deletions revealed that a fragment of only 45 amino acids retained CaM binding capability (Figure S8). According to the CaM target database (http://calcium.uhnres.utoronto.ca/ctdb), this amino acid sequence does not contain any of the known CaM-binding motifs. However, as shown in Figure 5c, it does contain a predicted coiled coil domain, and it is amphipathic, a property shared by almost all CBDs [61].

Previous studies showed that disruption of amphipathic properties of CBDs abolished CaM binding [41],[62], so we further defined the CBD of CBP60g using site-directed mutagenesis. Based on the helical wheel projection of the CBP60g CBD (Figure 5C), we mutated the codons for all four hydrophobic amino acids (three valines and one phenylalanine) on the hydrophobic side to create codons for hydrophilic amino acids (arginine or lysine). As controls we also mutated codons for two amino acids on the hydrophilic side of the CBP60g CBD and one amino acid just outside the CBD. These changes did not affect the amphipathic nature of the predicted helix. Figure 5D shows that loss of any of the four hydrophobic amino acids on the hydrophobic side of the CBD abolished CaM binding, while none of the other mutations had a detectable effect. Taken together, these experiments demonstrated that CBP60g is a CaM-binding protein, and defined the CBD in the N-terminus of the protein.

### CaM Binding Is Required for CBP60g Function in Disease Resistance and SA Accumulation

CaM binding often modulates protein function [40]. To investigate whether CaM binding affects the function of CBP60g in defense responses, we engineered transgenic plants carrying mutated CBP60g proteins that no longer bind CaM in the *cbp60g-1* mutant background. We then tested them for defects in limiting bacterial growth and SA accumulation. We transformed *cbp60g-1* mutant plants with modified genomic constructs including both CaM-binding (F41K) and non-CaM-binding (V28K, V29R) versions of CBP60g, which were fused to a c-Myc epitope tag at their C-termini. A wild-type version of the CBP60g c-Myc fusion construct (WT) was also made as a control. Primary transformants containing single copies of the transgenes were selected by qPCR, and their progeny were used for analyses. First, we tested expression of the modified proteins by immunoblotting using c-Myc antibody. None of the c-Myc fusion proteins were detected in untreated plants, but they were all present in plants inoculated with *Psm* ES4326. This was also true for the wild-type CBP60g c-Myc fusion construct (Figure S9A). Thus, the *Psm* ES4326-induced increase in the CBP60g transcript level is reflected in the protein level. We then measured bacterial growth and SA accumulation in the transgenic plants. Figure 6A shows that 2 days after inoculation with *Psm* ES4326, bacterial titers in transgenic lines carrying non-CaM-binding constructs were similar to the titers in *cbp60g-1*, while titers in transgenic lines carrying the CaM-binding construct were similar to those in wild-type plants. We assayed four additional independent transgenic lines for bacterial growth, yielding consistent results (Figure S9B). This shows that CaM binding is required for complementation of the enhanced disease susceptibility phenotype of *cbp60g-1*. We also measured free SA levels in leaves after treatment with flg22 or infection by *Psm* ES4326. Figure 6B shows that the non-CaM-binding proteins, V28K and V29R, failed to complement the SA accumulation defects of *cbp60g-1*, while the protein that did bind CaM, F41K, restored SA to wild-type levels. Collectively, these results demonstrate that CBP60g requires CaM binding for its function in disease resistance and MAMP-induced SA accumulation.

## Discussion

Our reverse-genetic study of CBP60g revealed that this gene is required for wild-type levels of resistance to the bacterial pathogens *Psm* ES4326 and *Pst* DC3000, indicating that it plays a role in plant defense. Expression profiling studies suggested a defect in activation of SA signaling during the MAMP response. SA assays proved that CBP60g contributes to MAMP-induced SA accumulation. We found that CaM binding is important for the role of CBP60g in defense signaling. In contrast to other members of the CBP60 family, the CaM-binding domain of CBP60g lies close to the N-terminus of the protein. CaM binding is needed for activation of the protein, as mutants that fail to bind CaM also fail to complement the SA and bacterial growth defects of loss-of-function mutants. Our work demonstrates that CBP60g constitutes a CaM-dependent link from MAMP signaling to activation of SA synthesis.

### The Role of CBP60g in MAMP Signaling

**Figure 7:**
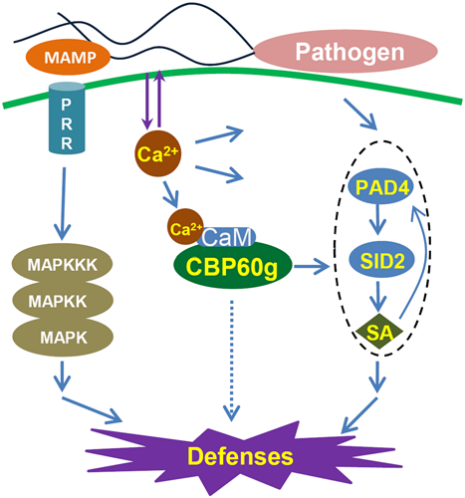
Model of CBP60g function in defense signaling. Binding of MAMPs by pattern recognition receptors initiates a MAPK signaling cascade that leads to activation of defense gene expression through WRKY transcription factors. MAMP recognition also induces production of reactive oxygen species by AtRBOHD, callose deposition, cytosolic Ca^2+^ flux, and *CBP60g* expression. CBP60g, when activated by CaM binding, positively regulates signaling leading to SA accumulation and defense gene expression.

Figure 7 shows a model of the position of CBP60g in the defense signaling network. Recognition of MAMPs such as bacterial flagellin by pathogen recognition receptors (PRRs) activates a MAP kinase cascade that in turn activates gene expression changes and ethylene production. MAMP recognition also triggers elevation of cytosolic Ca^2+^ concentration and activates production of reactive oxygen species (ROS) by AtrbohD. AtrbohD is required for deposition of callose. Recently, we found that MAMP signaling also activates SA production, and that activation of SA signaling by MAMPs is important for MAMP-induced resistance [33]. SA signaling is also activated in response to recognition of effectors by R genes (ETI). Infection by the virulent strain *Psm* ES4326 activates SA signaling strongly, and infection by *Pst* DC3000 activates it to a lesser degree [48]. It is not known whether this activation is due to a weak ETI response that does not result in a hypersensitive response, or to some other mode of pathogen recognition.

The increase in cytosolic Ca^2+^ triggered by MAMP recognition likely affects may aspects of defense signaling, as suggested by the multiple arrows leading out from Ca^2+^ in Figure 7. One of these aspects is activation of CBP60g through CaM binding, as we have shown that CaM binding is required for the functions of CBP60g in MAMP-induced SA accumulation and limiting growth of *Psm* ES4326. CBP60g contributes to MAMP-induced SA accumulation, as SA levels are reduced in *cbp60g* mutants. Following *Psm* ES4326 inoculation, an SA-accumulation defect was observed at nine but not 24 hours. SA accumulation at nine hours likely reflects MAMP signaling, so this data is consistent with the idea that CBP60g is involved in transducing a signal from the MAMP response to SA accumulation. It is likely that there are multiple routes to activation of SA accumulation, with different routes more or less important for different stimuli and/or at different times. This may explain our finding that CBP60g is important for SA accumulation during the MAMP response, but has little effect during the response to *Psm* ES4326.

While our results show that CBP60g constitutes part of the link between MAMP recognition and activation of SA signaling, we cannot yet determine at what point in the MAMP signaling cascade a signal is transferred to CBP60g. Similarly, the relationship between MAMP recognition and Ca^2+^ influx is unclear. These uncertainties are indicated by the absence of arrows between PRRs and the MAPK cascade on the one hand, and Ca2+ influx and CBP60g function on the other. Based on examination of the microarray data, we speculate that CBP60g and the MAPK cascade may act independently. As shown in Figure S10, at 3 and 9 hours after inoculation with *Pst* DC3000 *hrcC*, there is no overlap between genes whose expression is affected by *mpk3* and those whose expression is affected by *cbp60g*. If CBP60g function required MAPK activation, or vice versa, we would expect to see some commonly-affected genes. However, it is also possible that if we were able to study a *mpk3 mpk6* double mutant (MAPK3 and MAPK6 are partially redundant, and a double mutant is lethal), we might see a different result.

One might also ask at what point CBP60g function affects SA signaling. The signal coming from CBP60g must act upstream from SA synthesis, as SA levels are reduced in *cbp60g* mutants. PAD4 also contributes to SA levels, as *pad4* mutants have reduced SA after MAMP treatment and after *Psm* ES4326 infection [33]. Unlike *pad4*, *cbp60g* does not affect SA levels at late times after infection by *Psm* ES4326, and it does not have a substantial effect on gene expression 24 hours after infection [22],[48]. It may affect SA levels independently of PAD4, or it may act upstream of PAD4. This uncertainty is indicated by the dotted circle on the right in Figure 7. Among the mutants studied by expression profiling, the effect of *cbp60g* was most similar to that of *pad4*, and slightly less similar to that of *sid2*. This may be an indication that *cbp60g* acts upstream of *pad4* to activate SA signaling during the MAMP response.

### Attenuated MAMP-Induced SA Signaling May Explain the Enhanced Susceptibility of *cbp60g* Plants to *Psm* ES4326

*Psm* ES4326 is a strong inducer of SA synthesis [22],[48]. In turn, SA-dependent defense responses play a major role in limiting growth of this pathogen. Mutations that seriously compromise SA signaling, including *pad4*, *eds5*, *sid2*, and *npr1*, result in increases in bacterial growth on the order of 2–3 log_10_s [63]–[65]. In *cbp60g* mutants, we observed reduced SA production following MAMP treatments, and this was reflected in delayed SA accumulation in plants inoculated with *Psm* ES4326, evidenced by reduced SA levels nine hours after infection. Growth of *Psm* ES4326 was enhanced by about 10-fold in *cbp60g* mutants, a smaller effect than observed in canonical SA pathway mutants. Could the delay in SA accumulation be responsible for the enhanced pathogen growth? This seems possible. Responses to avirulent and virulent *P. syringae* strains were shown to be quite similar, with the major differences lying in the relative speed and amplitude of responses, rather than in qualitative effects [66]. Thus, a delay in launching a critical response such as SA signaling could well have a dramatic effect on resistance. Alternatively, CBP60g may have other defense response defects in addition to delayed SA accumulation, which we have not yet detected. These defects, combined with the delay in SA accumulation, may result in enhanced growth of *Psm* ES4326.

The effect of MAMP responses on resistance can be detected by pre-treating plants with flg22, and then inoculating with *Pst* DC3000 one day later. In wild-type plants, this results in a 3-log_10_ reduction in bacterial growth [15]. In *pad4* and *sid2* plants, this difference was reduced, with the effect of *sid2* being stronger than the effect of *pad4* [33]. We tested *cbp60g* mutants using this assay. While *Pst* DC3000 grew to higher titers in *cbp60g* mutants than in wild-type plants, the growth reduction due to flg22 pre-treatment was not significantly different in *cbp60g* and wild-type plants (Figure S11). MAMP-induced SA levels are higher in *cbp60g* mutants than in *pad4*, which are in turn higher than in *sid2*. It is likely that the reduction of SA in *cbp60g* plants is not sufficiently severe to compromise flg22-induced resistance. Similarly, systemic acquired resistance to *Psm* ES4326 was not affected in *cbp60g* mutants (Figure S12), suggesting that the reduction in SA produced in response to the *Psm* ES4325 pre-infection was not sufficiently severe to compromise SAR.

### CaM Binding is Required for the Function of CBP60g in Defense Signaling

We identified a CaM-binding domain near the N-terminus of CBP60g (Figure S1). This domain is predicted to form a basic, amphipathic helix, but is otherwise unlike known CaM-binding motifs. Plant CaM binding domains are known to be highly polymorphic. Previous studies have identified several motifs that are conserved in some CaM binding proteins. These include the 1–10 and 1–14 motifs described by Rhoads et al [67]; the 1–16 motif described by Osawa et al [68] and the IQ motif described by Cheney et al [69]. Our work adds another defined sequence to the known CaM-binding domains. Mechanisms by which CaM binding can modulate protein function include relieving auto-inhibition, remodeling active sites, and mediating dimerization [70]. The placement of the CaM-binding domain near the N-terminus is consistent with a role of CaM binding in relieving auto-inhibition or in promoting dimerization. It seems unlikely that it remodels an active site, as the central portions of CBP60 proteins show extensive conservation (Figure S1), yet other CBP60 proteins have C-terminal CaM-binding sites while CBP60g has an N-terminal site.

CaM binding is needed for activation of CBP60g function, as mutants lacking CaM binding activity could not complement the SA and pathogen growth defects of *cbp60g* insertion mutants. Thus, CBP60g is regulated at two levels, elevated mRNA and protein in response to pathogen attack, and Ca^2+^ in the form of CaM binding. Such a “double check” mechanism may suggest an adverse effect of initiating a CBP60g-dependent defense response. Indeed, we were unable to obtain plants expressing CBP60g under the control of the strong 35S promoter, suggesting that unregulated expression of CBP60g is deleterious.

### CBP60g Constitutes a Link between Ca^2+^ and SA Signaling

While there is abundant evidence that Ca^2+^ acts as a signal in the MAMP response, relatively little is known about the effect of this signal. In parsley cell cultures, production of phytoalexins in response to the MAMP Pep-13 requires Ca^2+^ influx [71]. Here, we provide evidence that Ca^2+^ affects activation of MAMP-induced SA signaling. CaM binds *cbp60g* only in the presence of Ca^2+^, and CaM binding is required for CBP60g to promote SA signaling. CBP60g thus links Ca^2+^ to SA signaling. This connection could constitute part of the system plants use to discriminate among pathogens, and between pathogens and beneficial or harmless microbes. Ca^2+^ has long been considered as a ubiquitous second messenger for many signaling cascades, including defense signaling [40],[70],[72]. The complexity of calcium patterns responding to different stimuli led to hypothesis that these patterns encode information that is relayed to downstream signaling components. Germinating spores of *Gigaspora margarita* (a beneficial soil fungus that forms a mutualistic association with its plant host) led to a single transient cytosolic Ca^2+^ elevation in soybean cell culture that lasted only 20 minutes [73]. Treatment with Rhizobium lipochitooligosaccharide nodulation factors led to rapid periodic cytosolic Ca^2+^ spikes in alfalfa root hairs without dramatically altering the basal cytosolic Ca^2+^ concentration [74]. In tobacco cell cultures flg22 led to biphasic cytosolic Ca^2+^ elevation that lasted several hours [75]. These studies suggested that the calcium signatures in beneficial host microbe interactions may differ from those of pathogenic ones [40]. Perhaps CBP60g is only activated in response to Ca^2+^ signatures characteristic of a pathogen attack, mediated by various CaM proteins in the plant.

## Materials and Methods

### Plant Genotypes, Growth Conditions, and Pathogen Inoculation

Wild type Columbia (Col-0), *pad4-1* (At3g52430) [76], *sid2-2* (At1g74710) [26], *fls2* (At5g46330; SAIL_691C4) [15], *pmr4-1* (At4g03550) [77], *mpk3* (At3g45640; SALK_151594) [78], *ein2-1* (AT5G03280) [79], *dde2-2* (AT5G42650) [55], *coi1-1* (AT2G39940) [80], *cbp60g-1* (At5g26920; SALK_023199), and *cbp60g-2* (GABI_075G12) Arabidopsis plants were grown on autoclaved BM2 Germinating Mix (Berger Inc., Quebec Canada) in a growth chamber at 22°C and a 12 hours photoperiod under 100 mM m^−2^ s^−1^ fluorescent illumination with 75% relative humidity. Plants were 4–5 weeks old at the time experiments were performed. *Psm* ES4326, *Pst* DC3000 and *Pst* DC3000 *hrcC^−^* strains were cultured at room temperature in King's B medium (protease peptone, 10 mg/ml; glycerol, 15 mg/ml; K_2_HPO_4_, 1.5 mg/ml; MgSO_4_, 5 mM, pH 7.0) with 50 µg/µl streptomycin (*Psm* ES4326) or 25 µg/µl rifampicin (*Pst* DC3000 and *hrcC^−^*). Flg22 peptide (EZBiolab Inc., IN, USA) was used at 1 µM.

### Bacterial Growth Assays

*Psm* ES4326, and *Pst* DC3000 suspensions in 5 mM MgSO_4_ of OD_600_ = 0.0002, and OD_600_ = 0.0001, respectively, were infiltrated into mature leaves using a needless syringe. Determination of bacterial titers was as described previously [81].

### Microarray Analysis

Expression profiling data for plants infected with *Psm* ES4326 was obtained and analyzed as part of the experiments described in [48]. The data is available from Gene Expression Omnibus (GEO; http://www.ncbi.nlm.nih.gov/geo/), accession number GSE11009. The data for *cbp60g* (SALK_023199) was not included in the Supplemental Tables for Wang et al., in press, because it was not discussed there. It is provided here as Table S1.

For the experiment using *Pst* DC3000 *hrcC*, mature leaves of 4.5-weeks-old plants were infiltrated with a bacterial suspension (OD_600_ = 0.05, 5×10^8^ cfu/ml), or water as mock treatment. Samples were collected 3 and 9 hours post inoculation. Three independent experiments were carried out. RNA was extracted using Trizol (Invitrogen, CA USA) described by Sato et al [50]. Expression profiles were analyzed in the R environment with the lme4 package after Stable genes Based Quantile (SBQ)-normalization [50]. For comparison of profiles between mock- and *Pst* DC3000 *hrcC*-infected Col-0 plants, the data were fitted to a 2-stage mixed effect linear model:
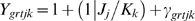


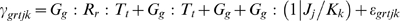
where 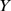, *G*, *R*, 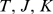, *γ*, and *ε* are log_2_-transformed expression level value, gene, treatment (mock- and hrcC-infected), time (3 and 9 hpi), experiment group, replicate, residual of the 1^st^ model, and residual of the 2^nd^ model. *G*, *R* and *T* are fixed effects, and 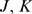 *γ*, and *ε* are random effects. The contrast of the 2^nd^ model was made to compare *Pst* DC3000 *hrcC*-infected and mock-infected values at each time for each gene. For comparison of profiles among different plant genotypes after *Pst* DC3000 *hrcC* infection, it was necessary to compensate for the fact that the data for *cbp60g-1* was obtained in a separate set of experiments from the experiments using the other mutants. The data from each of the two experiment groups were separately fitted to the above 2-stage model, except that *R* is genotype (8 genotypes) instead of treatment and that the model contains no 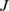. Using the fitted values for the samples common between pairs of experiment groups, calibration values that equalize the fitted values for the same genotype at each time point in different experiment groups were calculated. The calibration values were added to the initial SBQ-normalized data, and the calibrated data were fitted to the above 2-stage model except that *R* is genotype and that the first model includes an 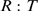 fixed effect. The contrast of the 2^nd^ model was made to compare the value of each genotype with that of Col-0 at each time for each gene.

### Cloning and site-specific mutagenesis of CBP60g

To make the complementation and transgenic site-specific mutagenesis constructs, the genomic coding sequence (with introns) of At5g26920 and an additional 1093 base pairs of DNA sequence upstream of its start codon was first amplified by polymerase chain reaction (PCR) using KOD Hot Start DNA Polymerase (Novagen, CA) and TA-cloned into the pCR8 vector following the manufacturer's protocol (Invitrogen, CA). It was then recombined into the Gateway-compatible pMDC123 binary vector [82] through the LR reaction (Invitrogen, CA). For testing CaM binding, mapping the CBP60g CBD, and identifying crucial amino acids of the CBP60g CBD, full length and various partial cDNA sequences of CBP60g (without the promoter or introns) was cloned into the pDEST15 vector (Invitrogen, CA) and expressed in *E. coli*. Site-specific mutagenesis of CBP60g was performed using the Phusion™ Site-Directed Mutagenesis Kit (New England Biolabs Inc., MA USA). For determination of CaM binding and production of transgenic plants carrying mutated versions of *CBP60g*, site-specific mutagenesis was carried out beginning with a full-length cDNA clone or a genomic clone, respectively, in pCR8. Cloning and mutagenesis primers used in these experiments are listed in Table S3. Arabidopsis transformation was carried out using *Agrobacterium tumefaciens* stain C58C1 as described by [83]. All cloned DNA sequences were verified by sequencing.

### Determination of Transgene Copy Number by Quantitative PCR

3–4 leaves from each 4-weeks-old transgenic plant were collected and homogenized in liquid nitrogen using a mortar and pestle. 0.5 ml of extraction buffer (100 mM Tris pH = 8.0, 50 mM EDTA pH = 8.0, 500 mM NaCl, and 10 mM β-mercaptoethanol) with 35 µl of 10% SDS was then added. Samples were incubated at 65°C for 10 minutes, and DNA was precipitated by adding 130 µl 5 M potassium acetate. Samples were then treated with 10 µ/ml RNase, ethanol-precipitated, washed and quantified before use. The copy number of the BAR transgene relative to that of single copy gene, *RMP1*, was determined by qPCR experiments according to Stahl et al. [84] using the SYBR Green JumpStart™ kit (SIGMA, MO USA) following the manufacturer's protocol. The thermal cycling program used was 94°C for 2 min, followed by 40 cycles of 94°C for 15 sec, 60°C for 1 min and 72°C for 1 min. Experimental readouts were obtained using ABI7500 Real Time PCR system (Applied Biosystems, Foster city, CA, USA). Copy number of transgenes was determined as described by Bubner et al [85]. Primers used in these experiments are listed in Table S3.

### Quantitative RT-PCR Analysis of Gene Expression

RNA was purified using Trizol (Invitrogen, CA USA). Quantitative RT-PCR experiments were carried out using an ABI7500 Real Time PCR system (Applied Biosystems, Foster city, CA, USA) and the SuperScript™ III Platinum® SYBR® Green One-Step qRT-PCR kit (Invitrogen, CA USA), following the manufacturer's protocol. The thermal cycling program was 50°C for 10 min, followed by 40 cycles of 95°C for 15 sec, 60°C for 1 min. *ACTIN2* (At3g18780) was used as the internal reference. Relative gene expression and probability values were calculated as described [33]. Primers used in these experiments are listed in Table S3.

### SA Assays

Mature leaves of 4.5 weeks-old plants were infiltrated with *Psm* ES4326 (OD_600_ = 0.01), *Pst* DC3000 *hrcC* (OD_600_ = 0.05) or 10 µM flg22 peptide. Determination of SA by solid-phase extraction, isotope dilution GC-MS, and data analysis were performed as described previously [33].

### Callose Quantification

Four weeks old plants were injected with 1 µm of flg22 suspension, and samples were collected 12 hours later. Infiltrated leaves were cleared overnight in alcoholic lactophenol (95% ethanol: lactophenol = 2∶1, lactophenol was made by mixing equal volumes of phenol, glycerol, lactic acid and water). Samples were then rinsed in 50% ethanol and then in water. Cleared leaves were stained with 0.01% aniline blue in 0.15 M phosphate buffer (pH = 9.5). Callose deposits were visualized under ultraviolet illumination using a Nikon Eclipse E600 microscope. Four pictures of different areas were taken of each leaf and callose deposits were counted using the “analyze particles” function of ImageJ (http://rsb.info.nih.gov/ij/). Six leaves were analyzed for each genotype, and three independent experiments were performed. P values were calculated using the Mann-Whitney U-test.

### Protein Expression in *E. coli*

Deletion and site-specific mutagenesis constructs of CBP60g were cloned into the pDEST15 plasmid vector, which creates N-terminal fusions to GST (Invitrogen, CA USA). They were then introduced into competent *E. coli* BL21(DE3) pLysS (Invitrogen) by electroporation. Colonies were selected on plates containing chloramphenicol (34 µg/ml) and ampicillin (50 LB µg/ml) plates. 200-µl aliquots of 2-ml overnight cultures were added to 4 ml of liquid LB medium containing 50 µg/ml chloramphenicol and 100 µg/ml ampicillin. They were then incubated at 37°C for 2 hr (OD_600_≈0.4) before addition of 20 µl of 200 mM IPTG. After a further 2 hr incubation at 37°C, samples were collected by centrifugation, washed with water, and resuspended in lysis buffer (50 mM potassium phosphate pH = 7.8, 400 mM NaCl, 100 mM KCl, 10% glycerol, 0.5% Triton X-100, 10 mM imidazol). Prior to loading on SDS gels, samples were frozen and thawed three times with liquid nitrogen and a 45°C water bath, and then mixed with same volume of 2× SDS-PAGE sample buffer (0.125 M Tris-HCL pH = 6.8, 20% glycerol, 4% β-mercaptoethanol, 0.2% bromophenol blue, 4% SDS).

### Immunoblots and CaM Binding Assays

Protein samples in 1× SDS-PAGE running buffer were separated on 8% acrylamide SDS gels and blotted to PVDF membranes according to the manufacturer's instructions (Bio-Rad). Membranes were incubated with 5% milk and then washed with TBST buffer (per liter: 2.423 g Tris-HCl, 8 g NaCl, and 0.1 ml Tween-20) before incubating with 10 µl of 0.25 mg/ml Rabbit monoclonal anti-GST antibody (Invitrogen) in 20 ml of TBST buffer. They were then washed three times with TBST, probed with anti-rabbit IgG conjugated alkaline phosphatase (AP) (Promega, CA), and visualized by incubating with 20 ml BCIP/NBT liquid substrate (Sigma). CaM binding assays were carried out using the Affinity® CBP Fusion Protein Detection Kit from Stratagene following the manufacturer's instructions. Some CaM assays were performed in the presence of 0.05 M ethylene glycol tetraacetic acid (EGTA) in TBST instead of 1 mM CaCl_2_, in order to test the Ca^2+^-dependence of CaM binding.

### Microarray data accession number

The original (.gpr) and normalized data files for the microarray analysis are available from Gene Expression Omnibus (http://www.ncbi.nlm.nih.gov/geo/) as GSE14237.

## Supporting Information

Figure S1 Multiple sequence alignment of Arabidopsis CBP60 proteins. Coding sequences of Arabidopsis CBP60 proteins were aligned using Multalin with default settings (http://bioinfo.genopole-toulouse.prd.fr/multalin/multalin.html). Red colored amino acids: consensus value>90%, blue colored amino acids: consensus value>50%. Underlined region A indicates the experimentally determined CBD of CBP60g, underlined region B indicates conserved CBDs of CBP60a, CBP60b, CBP60c, CBP60d, and CBP60e.
(9.45 MB TIF)

Figure S2 Bacterial growth in plants treated for SA measurement. Bacterial growth assays using *Psm* ES4326 (inoculation dosage: OD_600_ = 0.01). Each bar at 0, 9 and 24 hours represents data from 16 replicates. Error bars represent standard deviation from 16 samples. Comparisons were made between Col-0 and mutants at all time points using the two-tailed Mann-Whitney U-test. No P values smaller than 0.05 were found. This experiment was repeated three times, and similar results were obtained.
(1.60 MB TIF)

Figure S3 Free SA levels following inoculation with *Psm* ES4326 *avrRpt2*. Plants were inoculated with *Psm* ES4326 *avrRpt2* (inoculation dosage: OD_600_ = 0.002). Each bar represents data from 2 independent experiments. Each sample consisted of a pool of six infected leaves. Data were analyzed by ANOVA. Error bars represent standard error. There were no differences between Col and *cbp60g* at q<0.05. SA levels in *sid2* were significantly lower than in Col at all time points, in both mock and *Psm* ES4326 *avrRpt2*-inoculated samples (q<0.001).
(0.83 MB TIF)

Figure S4 Measurement of flg22-induced ROS. Oxidative burst induced by 10 µM flg22, measured as relative luminescence units (RLU). Flg22 was added at the beginning of the measurement. Each line represents the average of three replicates, each measured at 1 minute intervals. Student's T test showed no significant difference among *cbp60g* mutants and wild type control. Mutant *fls2* was used as a negative control that does not generate ROS in response to flg22 treatment.
(1.41 MB TIF)

Figure S5 Measurement of flg22-induced callose deposition. Aniline blue staining of callose deposits 12 hours after flg22 infiltration. The bar graph represents the average number of callose deposits observed per square millimeter. Error bars are standard deviation of 24 measurements, 4 from each of 6 leaves per genotype. Comparison between both *cbp60g* mutants and Col-0 were done using two-tailed Mann-Whitney U-test. No p-values were smaller than 0.05. Mutant *pmr4* was used as a negative control that does not produce callose deposits. This experiment was repeated four times, and similar results were obtained.
(4.59 MB TIF)

Figure S6 Inhibition of seedling growth by flg22 treatment. Flg22-induced growth inhibition was measured as described by Suarez-Rodriguez et al [86]. Each bar represents the mean weight of one seedling. Data were obtained in three independent experiments, each consisting of 12 replicates per sample type. Means and standard error (error bars) were calculated by ANOVA. 1 µM of flg22 was used to induce growth inhibition. Asterisks: p<0.001. Comparisons were made between mutants and wild-type within the same treatment.
(0.97 MB TIF)

Figure S7 CaM binding to CBP60g requires Ca^2+^. (A) Detection of calmodulin binding using washing buffer lacking CaCl_2_ and containing 5 mM EGTA (B) Detection of GST-fusion proteins using anti-GST antibody under the same conditions used in (A). The protein samples used were from the same preparations as those used in Figure 5.
(0.44 MB TIF)

Figure S8 Mapping of AtCBP60g CBD. A GST fusion containing the first 45 amino acids of CBP60g was detected using GST antibody and assayed for CaM binding. The top panel shows the immunoblot result with GST antibody, the bottom panel shows CaM binding results. (+IPTG: protein crude extract after IPTG induction, −IPTG: protein extract without IPTG added).
(3.88 MB TIF)

Figure S9 Measurement of bacterial growth in *cbp60g* transgenic lines. (A) Presence of modified CBP60g proteins in the *cbp60g-1* background. The upper panel shows the immunoblot results using anti-c-Myc antibody; the lower panel shows the large subunit of the Ribulose-1,5-bisphosphate carboxylase/oxygenase (Rubisco) stained with Ponceau S as a measurement of the total protein loaded onto each lane. M indicates mock inoculated, P indicates *Psm* ES4326 inoculated. (B) Bacterial growth measurement in Col-0, *cbp60g-1* and transgenic lines expressing altered CBP60g protein. Each bar represents the mean of 16 replicates and error bars represent standard deviations. P values were calculated by two-tailed Mann-Whitney U-test between Col-0 and mutants. Asterisks indicate p<0.05. The experiment was repeated twice, and similar results were obtained.
(4.07 MB TIF)

Figure S10 Lack of overlap between sets of genes affected by *mpk3* and *cbp60g*. Circles indicate sets of genes with significantly different (q<0.05) expression levels in *mpk3* or *cbp60g*, compared to wild-type plants. Data is from Table S2.
(1.49 MB TIF)

Figure S11 Measurement of *Pst* DC3000 growth after flg22 treatment. Growth of Pst DC3000 was measured in plants pre-treated with flg22 or water (mock). Each bar represents data from 32 replicates pooled from two independent experiments. Standard errors and p values were calculated by ANOVA.
(1.71 MB TIF)

Figure S12 Systemic acquired resistance in *cbp60g* mutants. The experiment was carried out as described by Mishina and Zeier [87]. (A) Three lower leaves of each plant were inoculated with either H_2_O or *Psm* ES4326 (O.D._600_ = 0.02) two days before inoculating two upper leaves with *Psm* ES4326 (O.D._600_ = 0.0001). Bacterial titers in the upper leaves were determined 0 and 2 days after the second inoculation. Each bar at 0 or 2 days represents data from 4 or 16 replicates, obtained in each of three independent experiments, respectively. Bars represent means and standard errors calculated by ANOVA. Asterisks, p<0.05; two asterisks, p<0.01. (B) Bar graph showing differences between water and *Psm* pretreated samples for each genotype.
(1.20 MB TIF)

Table S1 Expression profile of *cbp60g-1* at 24 hours after inoculation of *Psm* ES4326.
(0.09 MB XLS)

Table S2 Expression profiles at 3 and 9 hours after inoculation of *Pst* DC3000 *hrcC*.
(0.49 MB XLS)

Table S3 List of primers used.
(0.02 MB XLS)
