# Supplementary figures and images for: Arabidopsis CaM Binding Protein CBP60g Contributes to MAMP-Induced SA Accumulation and Is Involved in Disease Resistance against Pseudomonas syringae
Source: PLoS Pathog. 2009 Feb 13;5(2):e1000301. doi: 10.1371/journal.ppat.1000301 (PMC2633612; doi:10.1371/journal.ppat.1000301)

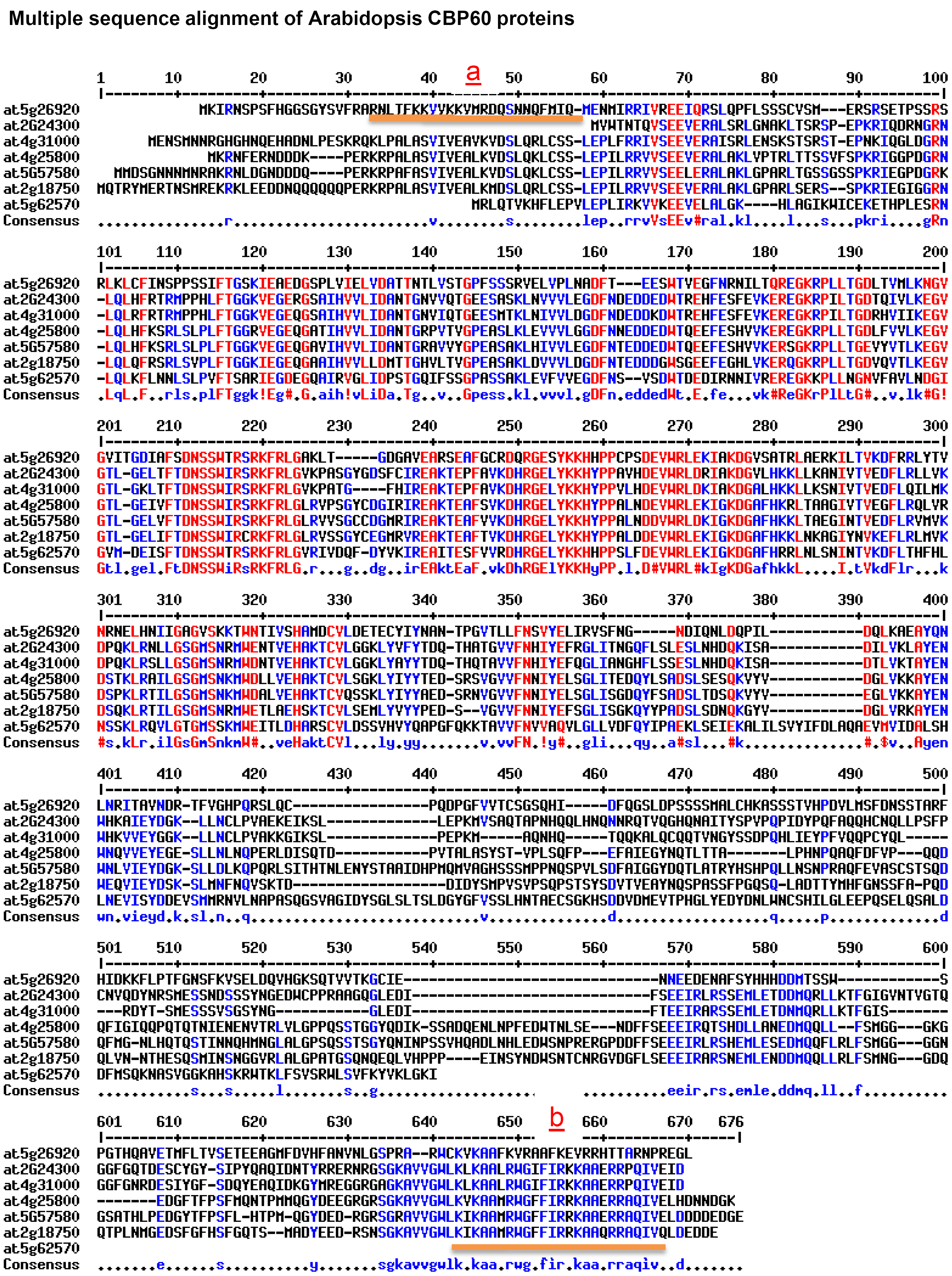

Supplement: Figure S1 — Multiple sequence alignment of Arabidopsis CBP60 proteins. Coding sequences of Arabidopsis CBP60 proteins were aligned using Multalin with default settings (http://bioinfo.genopole-toulouse.prd.fr/multalin/multalin.html). Red colored amino acids: consensus value>90%, blue colored amino acids: consensus value>50%. Underlined region A indicates the experimentally determined CBD of CBP60g, underlined region B indicates conserved CBDs of CBP60a, CBP60b, CBP60c, CBP60d, and CBP60e. (9.45 MB TIF) [file ppat.1000301.s001.tif]

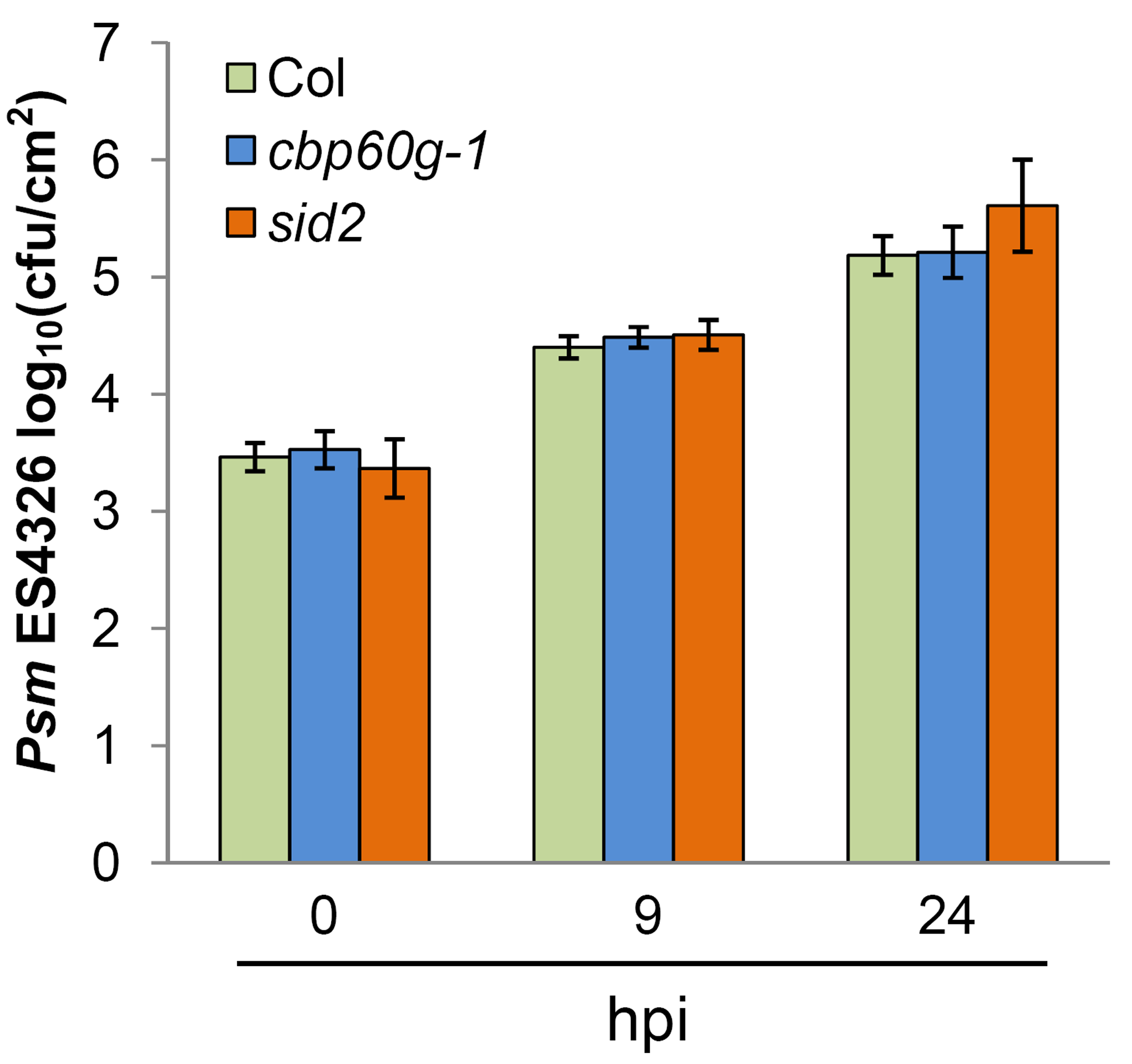

Supplement: Figure S2 — Bacterial growth in plants treated for SA measurement. Bacterial growth assays using Psm ES4326 (inoculation dosage: OD600 = 0.01). Each bar at 0, 9 and 24 hours represents data from 16 replicates. Error bars represent standard deviation from 16 samples. Comparisons were made between Col-0 and mutants at all time points using the two-tailed Mann-Whitney U-test. No P values smaller than 0.05 were found. This experiment was repeated three times, and similar results were obtained. (1.60 MB TIF) [file ppat.1000301.s002.tif]

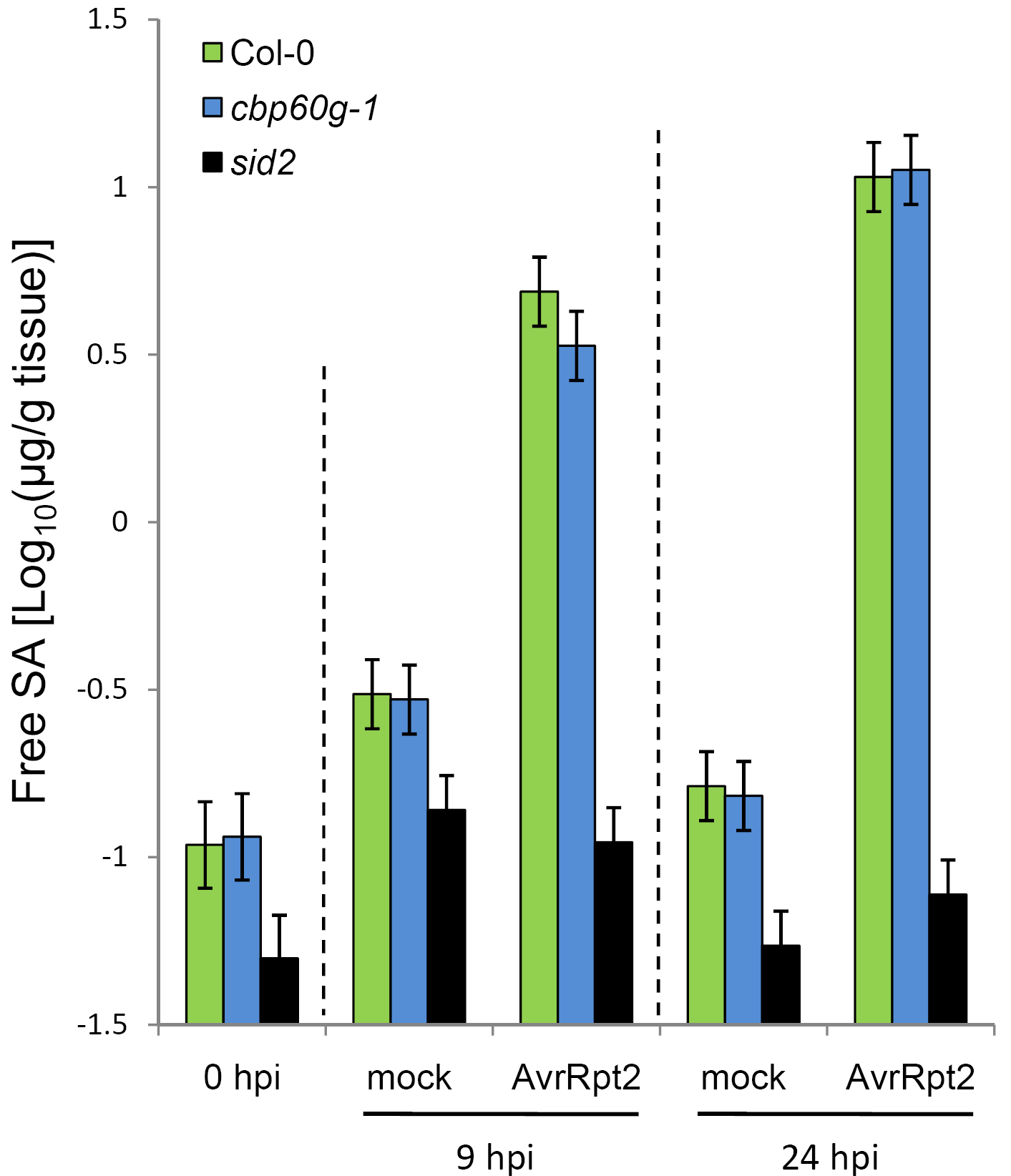

Supplement: Figure S3 — Free SA levels following inoculation with Psm ES4326 avrRpt2. Plants were inoculated with Psm ES4326 avrRpt2 (inoculation dosage: OD600 = 0.002). Each bar represents data from 2 independent experiments. Each sample consisted of a pool of six infected leaves. Data were analyzed by ANOVA. Error bars represent standard error. There were no differences between Col and cbp60g at q<0.05. SA levels in sid2 were significantly lower than in Col at all time points, in both mock and Psm ES4326 avrRpt2-inoculated samples (q<0.001). (0.83 MB TIF) [file ppat.1000301.s003.tif]

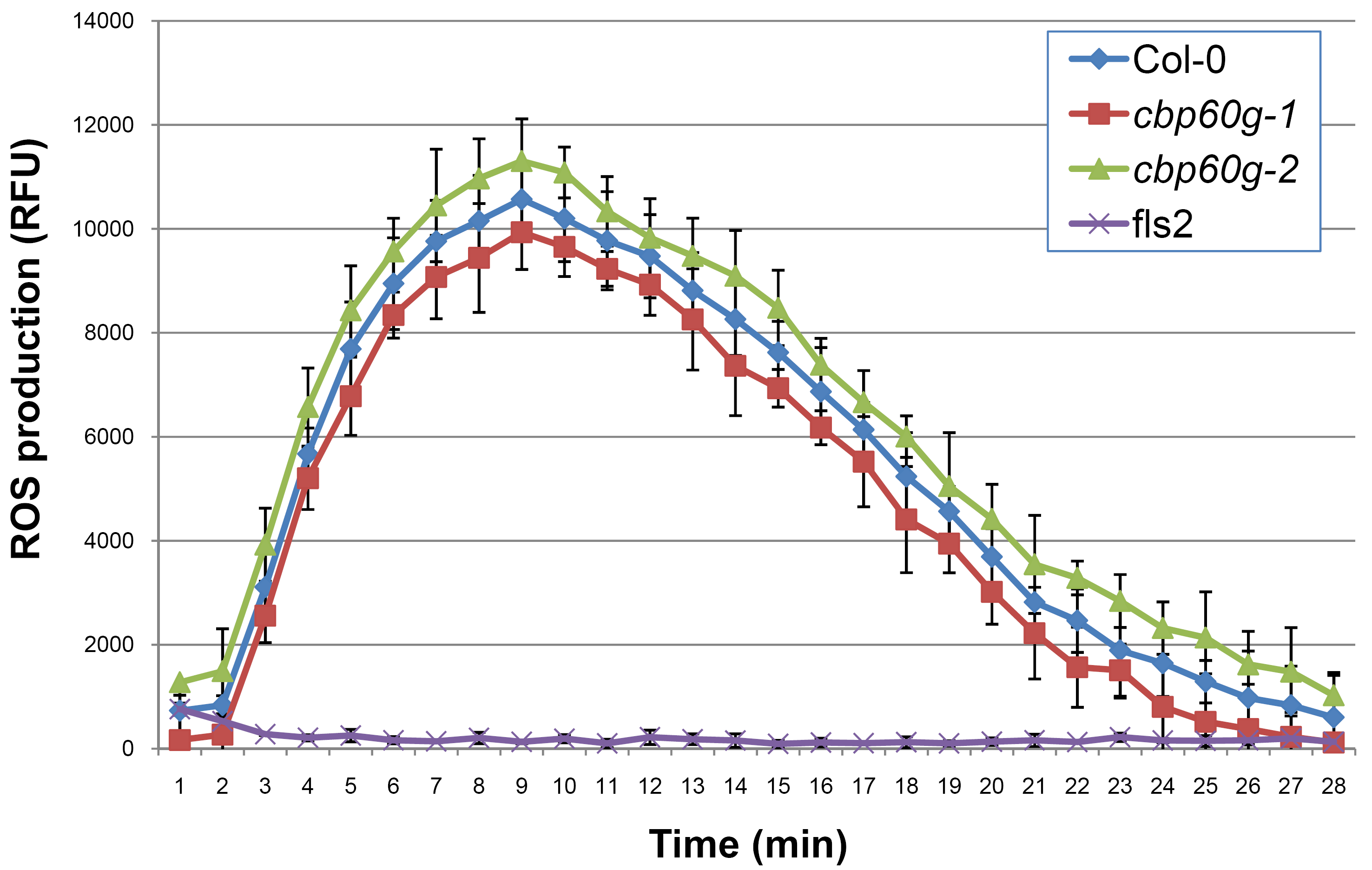

Supplement: Figure S4 — Measurement of flg22-induced ROS. Oxidative burst induced by 10 µM flg22, measured as relative luminescence units (RLU). Flg22 was added at the beginning of the measurement. Each line represents the average of three replicates, each measured at 1 minute intervals. Student's T test showed no significant difference among cbp60g mutants and wild type control. Mutant fls2 was used as a negative control that does not generate ROS in response to flg22 treatment. (1.41 MB TIF) [file ppat.1000301.s004.tif]

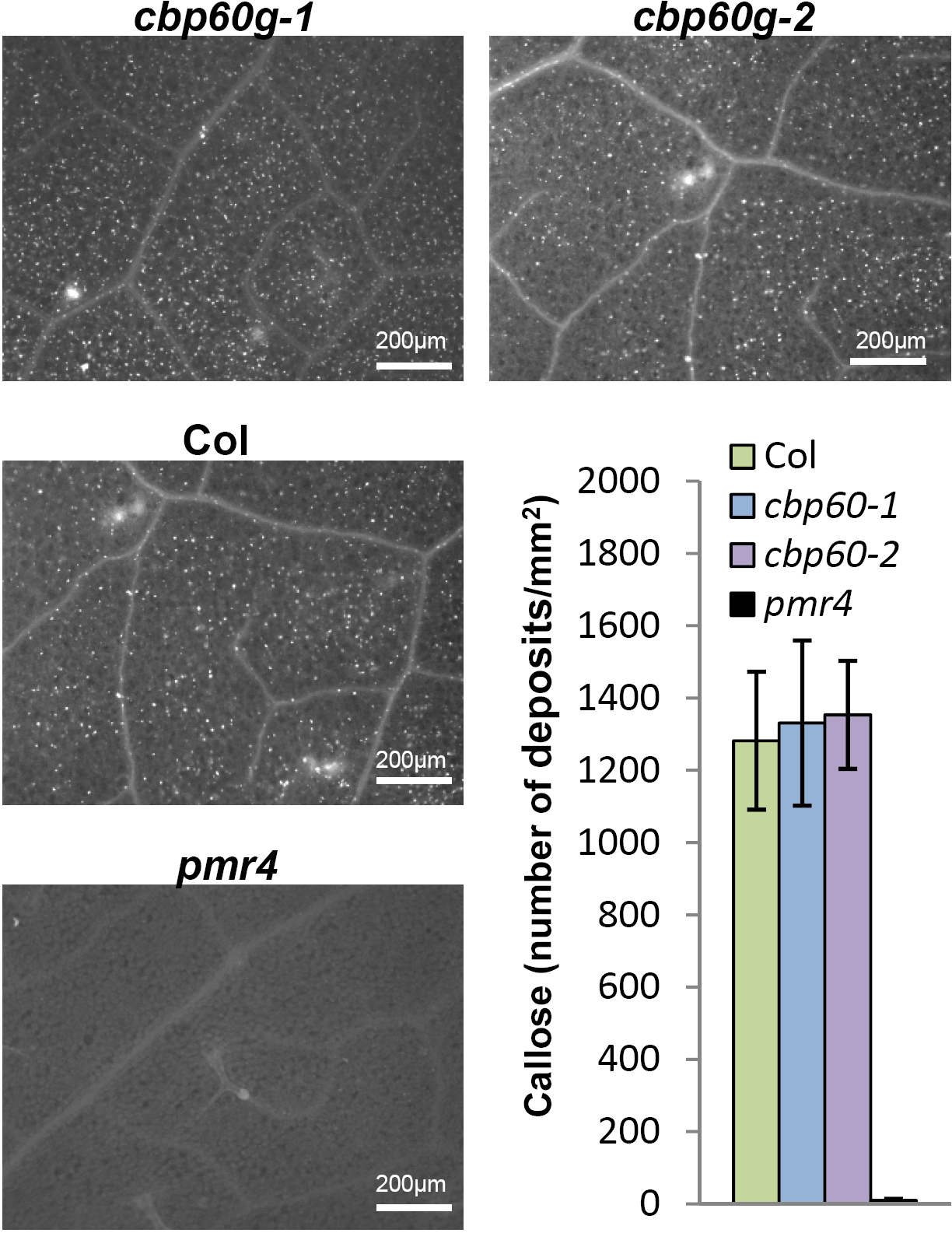

Supplement: Figure S5 — Measurement of flg22-induced callose deposition. Aniline blue staining of callose deposits 12 hours after flg22 infiltration. The bar graph represents the average number of callose deposits observed per square millimeter. Error bars are standard deviation of 24 measurements, 4 from each of 6 leaves per genotype. Comparison between both cbp60g mutants and Col-0 were done using two-tailed Mann-Whitney U-test. No p-values were smaller than 0.05. Mutant pmr4 was used as a negative control that does not produce callose deposits. This experiment was repeated four times, and similar results were obtained. (4.59 MB TIF) [file ppat.1000301.s005.tif]

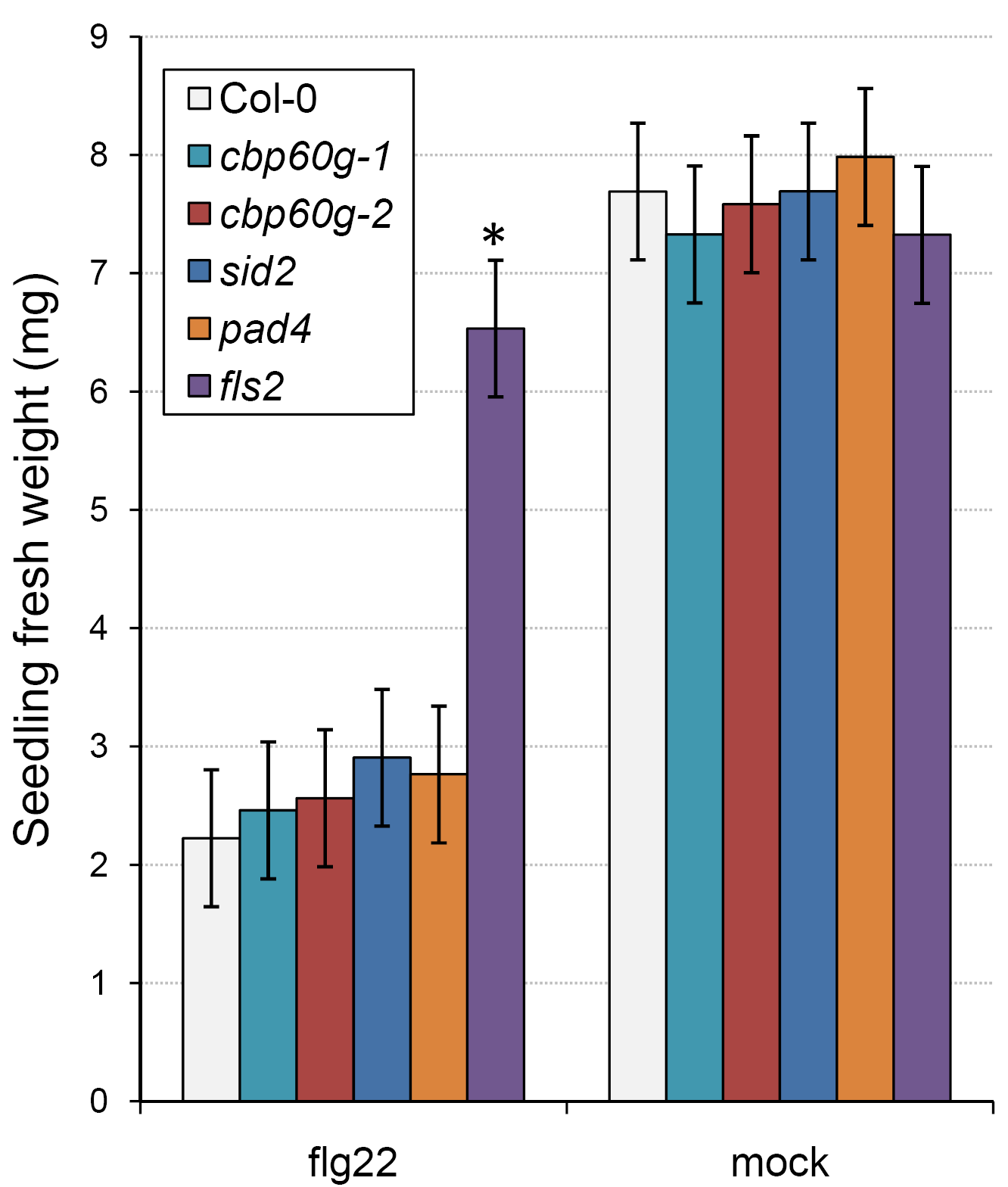

Supplement: Figure S6 — Inhibition of seedling growth by flg22 treatment. Flg22-induced growth inhibition was measured as described by Suarez-Rodriguez et al [86]. Each bar represents the mean weight of one seedling. Data were obtained in three independent experiments, each consisting of 12 replicates per sample type. Means and standard error (error bars) were calculated by ANOVA. 1 µM of flg22 was used to induce growth inhibition. Asterisks: p<0.001. Comparisons were made between mutants and wild-type within the same treatment. (0.97 MB TIF) [file ppat.1000301.s006.tif]

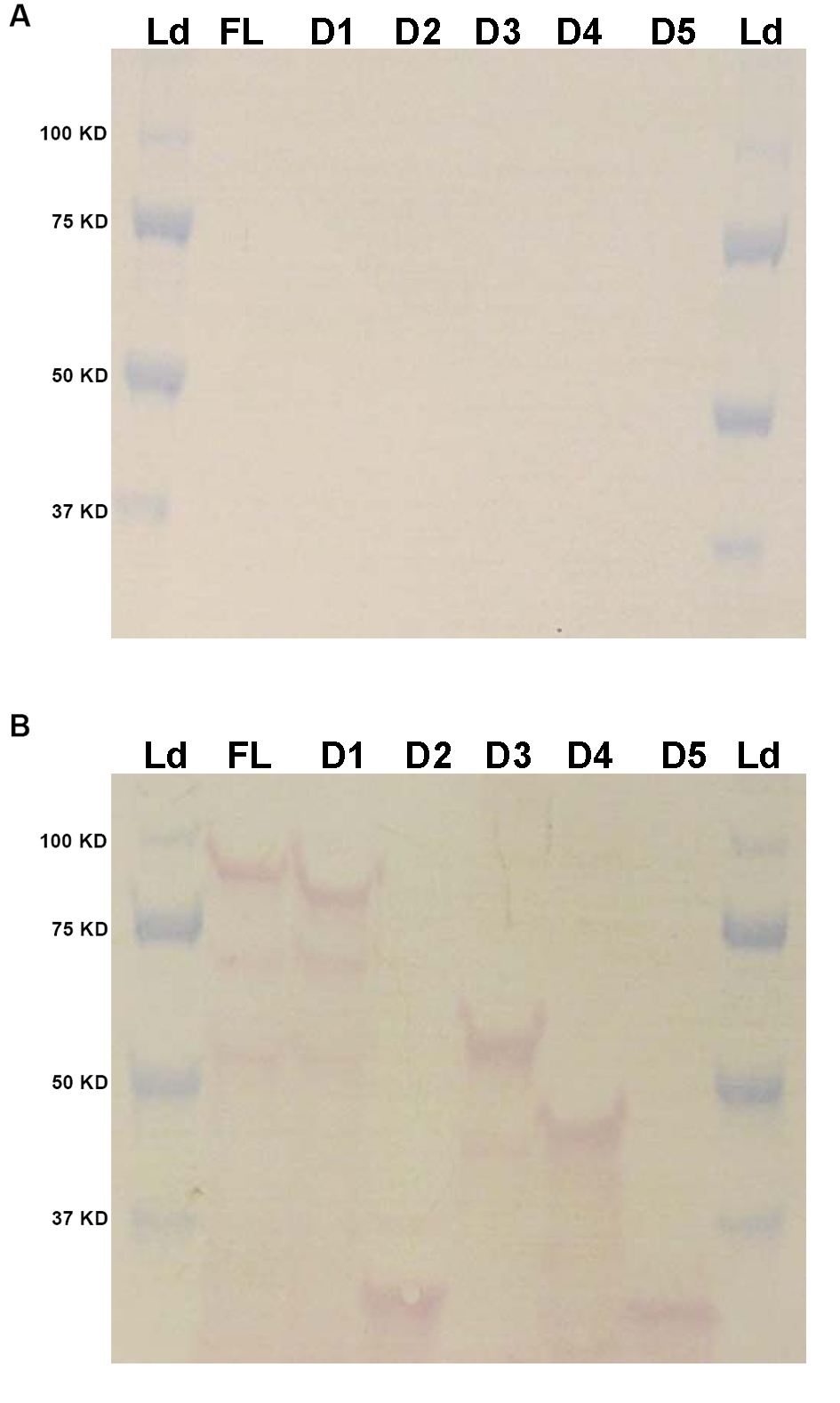

Supplement: Figure S7 — CaM binding to CBP60g requires Ca2+. (A) Detection of calmodulin binding using washing buffer lacking CaCl2 and containing 5 mM EGTA (B) Detection of GST-fusion proteins using anti-GST antibody under the same conditions used in (A). The protein samples used were from the same preparations as those used in Figure 5. (0.44 MB TIF) [file ppat.1000301.s007.tif]

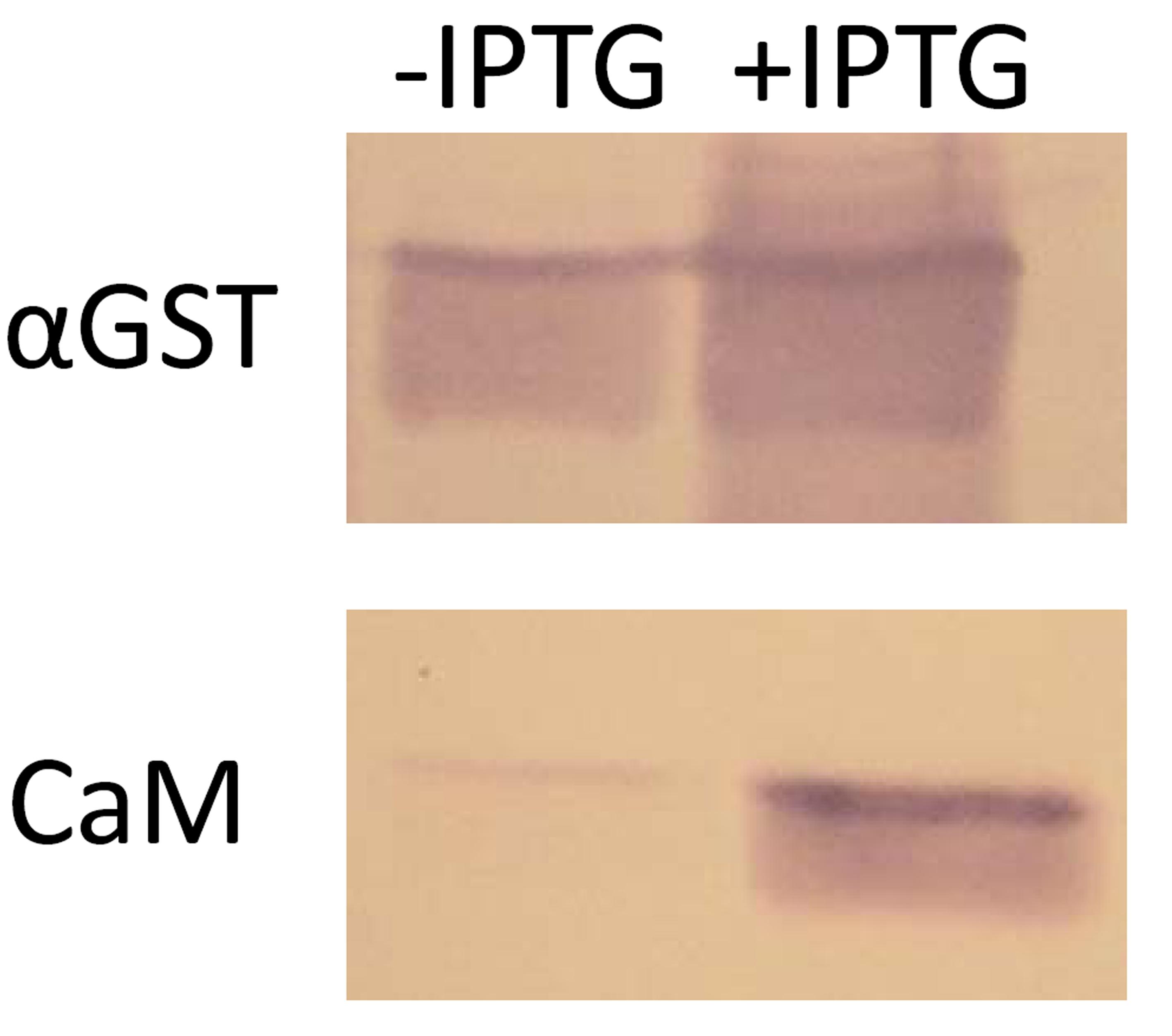

Supplement: Figure S8 — Mapping of AtCBP60g CBD. A GST fusion containing the first 45 amino acids of CBP60g was detected using GST antibody and assayed for CaM binding. The top panel shows the immunoblot result with GST antibody, the bottom panel shows CaM binding results. (+IPTG: protein crude extract after IPTG induction, −IPTG: protein extract without IPTG added). (3.88 MB TIF) [file ppat.1000301.s008.tif]

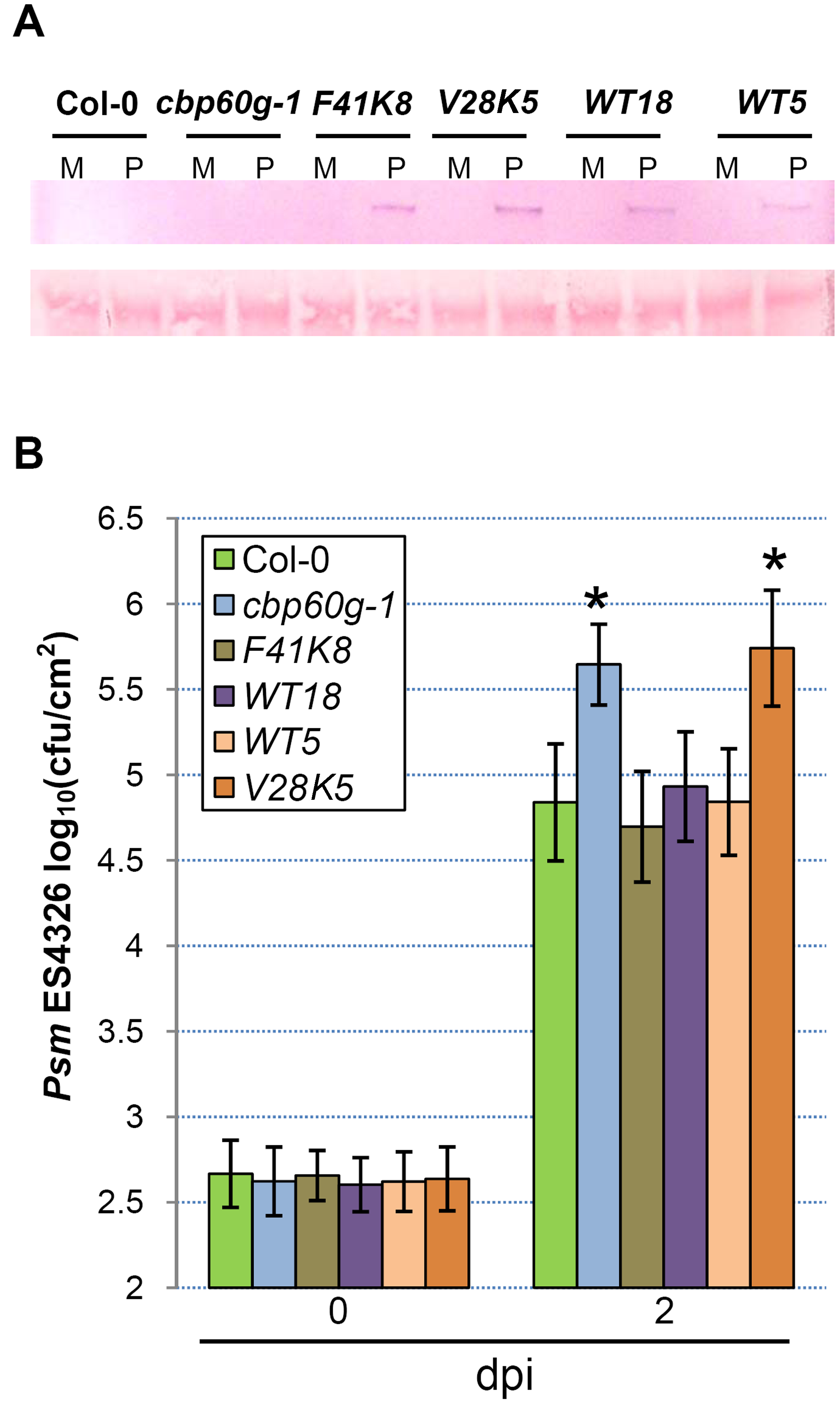

Supplement: Figure S9 — Measurement of bacterial growth in cbp60g transgenic lines. (A) Presence of modified CBP60g proteins in the cbp60g-1 background. The upper panel shows the immunoblot results using anti-c-Myc antibody; the lower panel shows the large subunit of the Ribulose-1,5-bisphosphate carboxylase/oxygenase (Rubisco) stained with Ponceau S as a measurement of the total protein loaded onto each lane. M indicates mock inoculated, P indicates Psm ES4326 inoculated. (B) Bacterial growth measurement in Col-0, cbp60g-1 and transgenic lines expressing altered CBP60g protein. Each bar represents the mean of 16 replicates and error bars represent standard deviations. P values were calculated by two-tailed Mann-Whitney U-test between Col-0 and mutants. Asterisks indicate p<0.05. The experiment was repeated twice, and similar results were obtained. (4.07 MB TIF) [file ppat.1000301.s009.tif]

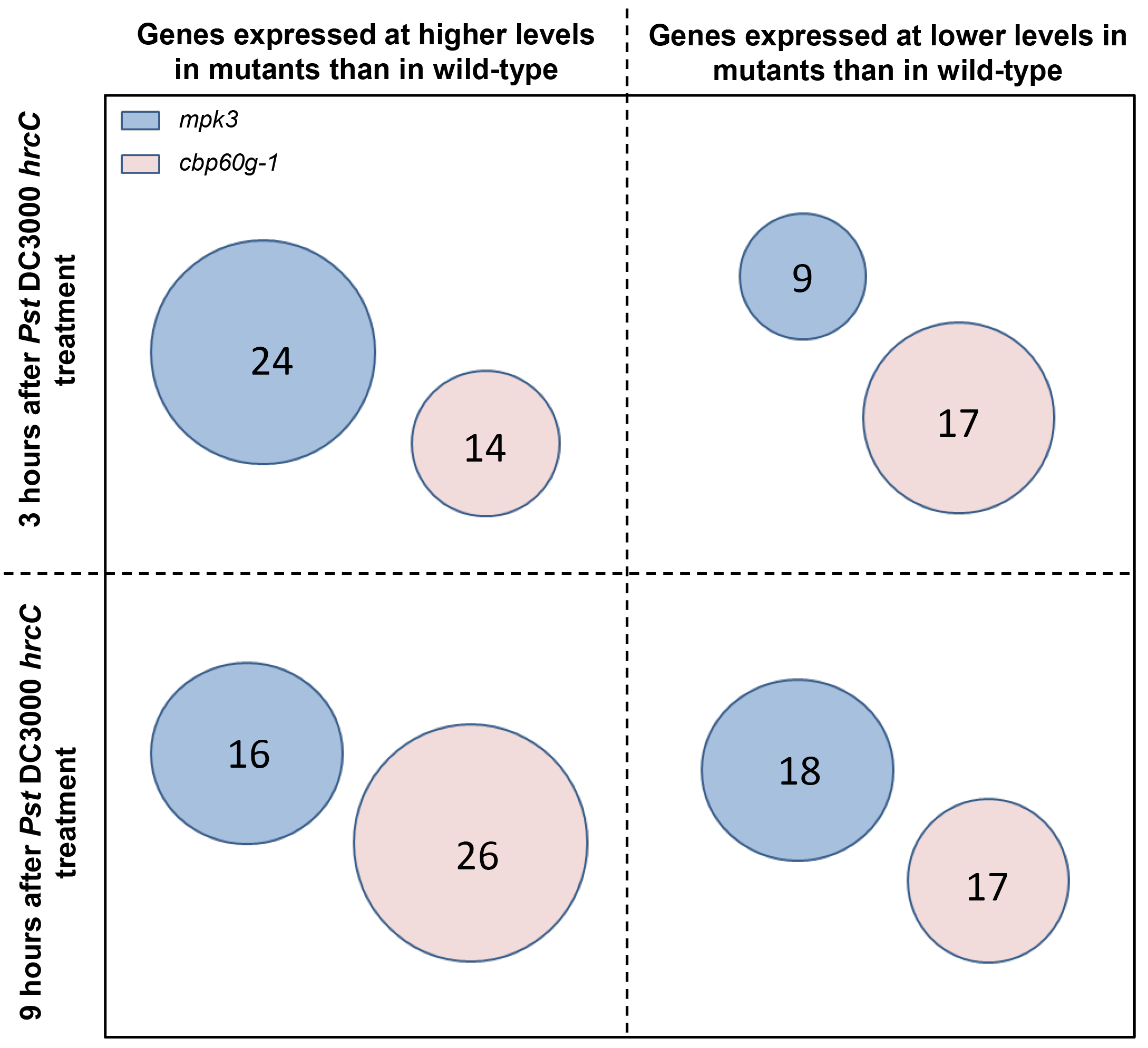

Supplement: Figure S10 — Lack of overlap between sets of genes affected by mpk3 and cbp60g. Circles indicate sets of genes with significantly different (q<0.05) expression levels in mpk3 or cbp60g, compared to wild-type plants. Data is from Table S2. (1.49 MB TIF) [file ppat.1000301.s010.tif]

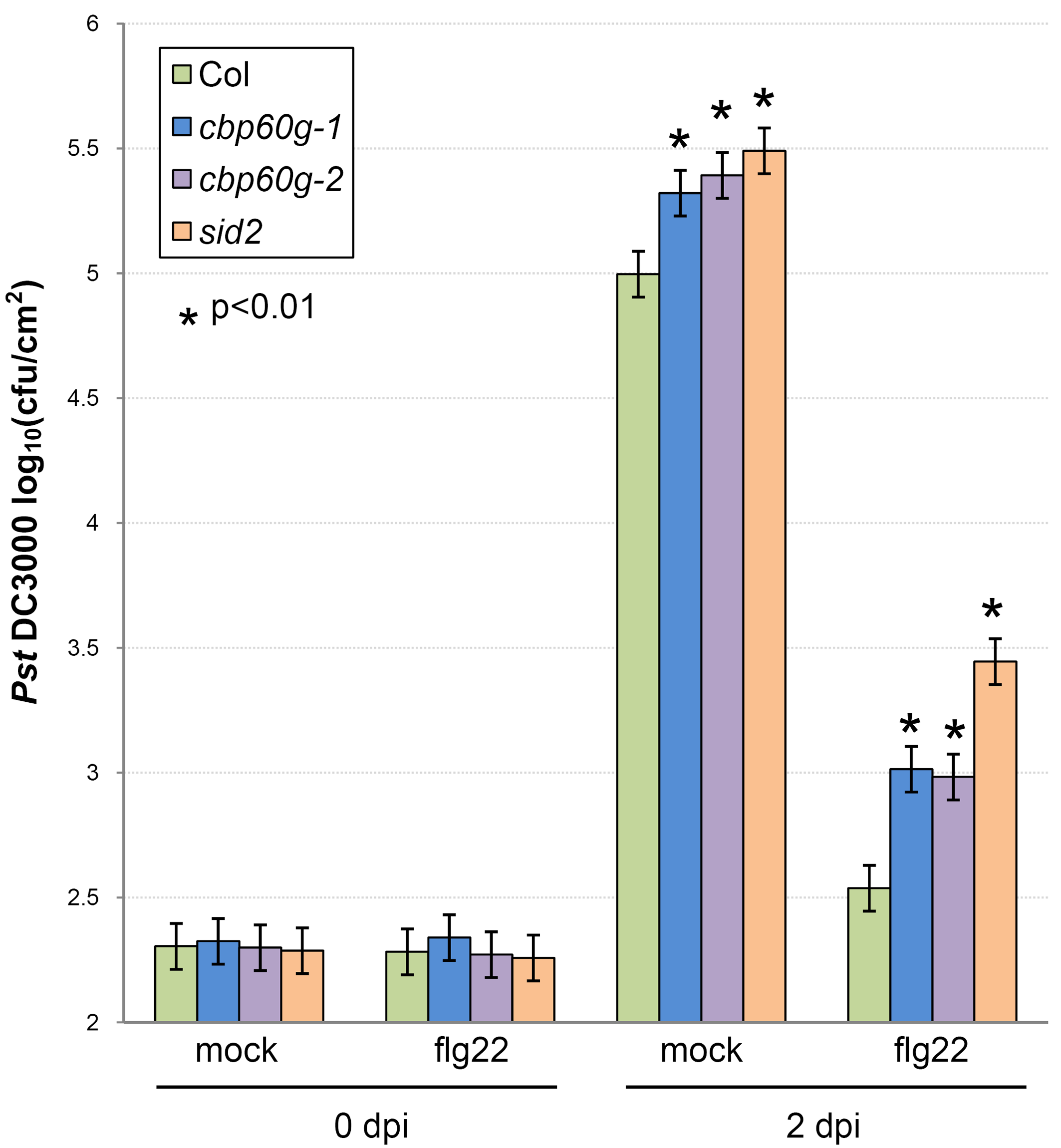

Supplement: Figure S11 — Measurement of Pst DC3000 growth after flg22 treatment. Growth of Pst DC3000 was measured in plants pre-treated with flg22 or water (mock). Each bar represents data from 32 replicates pooled from two independent experiments. Standard errors and p values were calculated by ANOVA. (1.71 MB TIF) [file ppat.1000301.s011.tif]

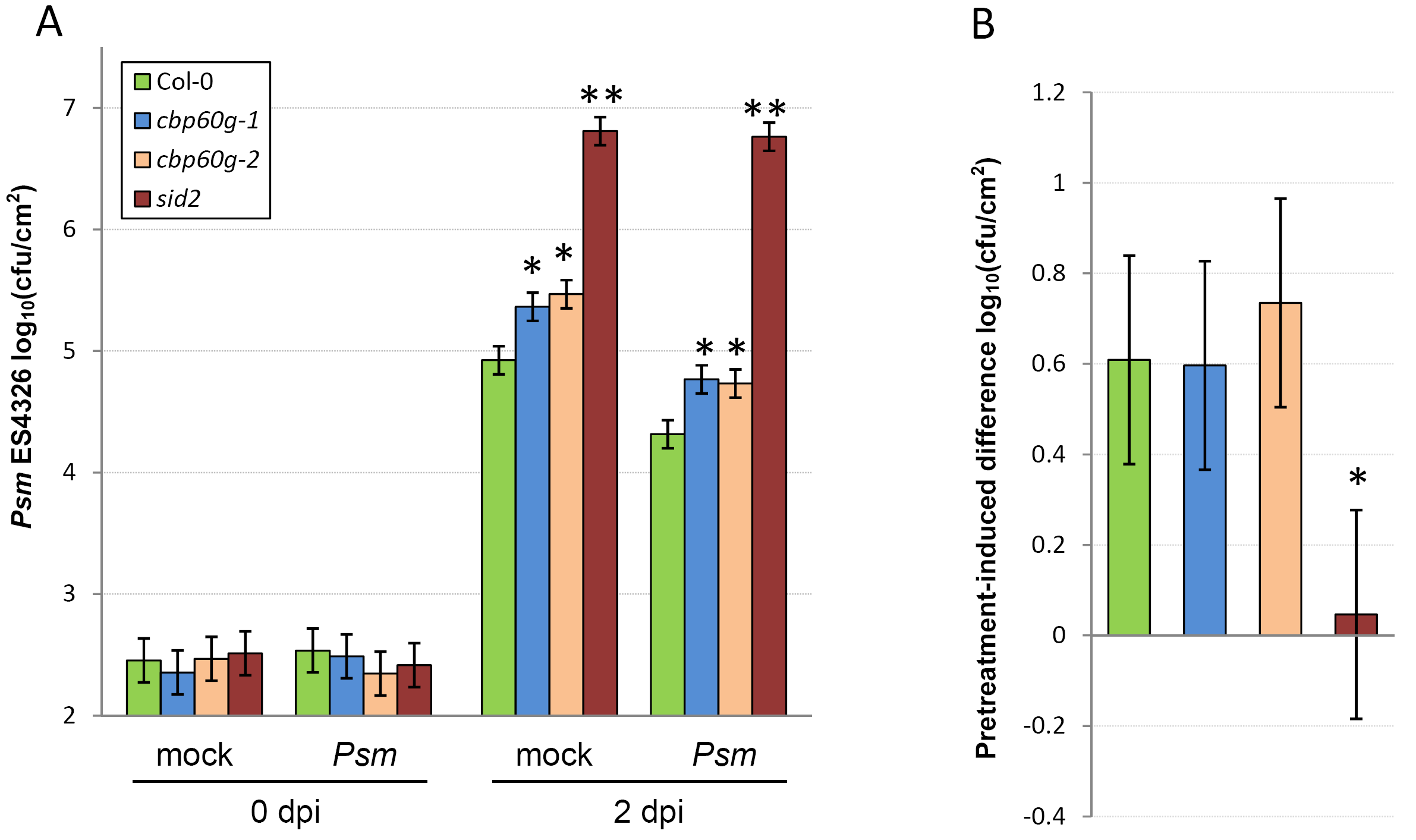

Supplement: Figure S12 — Systemic acquired resistance in cbp60g mutants. The experiment was carried out as described by Mishina and Zeier [87]. (A) Three lower leaves of each plant were inoculated with either H2O or Psm ES4326 (O.D.600 = 0.02) two days before inoculating two upper leaves with Psm ES4326 (O.D.600 = 0.0001). Bacterial titers in the upper leaves were determined 0 and 2 days after the second inoculation. Each bar at 0 or 2 days represents data from 4 or 16 replicates, obtained in each of three independent experiments, respectively. Bars represent means and standard errors calculated by ANOVA. Asterisks, p<0.05; two asterisks, p<0.01. (B) Bar graph showing differences between water and Psm pretreated samples for each genotype. (1.20 MB TIF) [file ppat.1000301.s012.tif]
